# *Drosophila* TIM Binds Importin α1, and Acts as an Adapter to Transport PER to the Nucleus

**DOI:** 10.1371/journal.pgen.1004974

**Published:** 2015-02-12

**Authors:** A. Reum Jang, Katarina Moravcevic, Lino Saez, Michael W. Young, Amita Sehgal

**Affiliations:** 1 Howard Hughes Medical Institute, Perelman School of Medicine at the University of Pennsylvania, Philadelphia, Pennsylvania, United States of America; 2 Laboratory of Genetics, The Rockefeller University, New York, New York, United States of America; Charité - Universitätsmedizin Berlin, GERMANY

## Abstract

Regulated nuclear entry of clock proteins is a conserved feature of eukaryotic circadian clocks and serves to separate the phase of mRNA activation from mRNA repression in the molecular feedback loop. In *Drosophila*, nuclear entry of the clock proteins, PERIOD (PER) and TIMELESS (TIM), is tightly controlled, and impairments of this process produce profound behavioral phenotypes. We report here that nuclear entry of PER-TIM in clock cells, and consequently behavioral rhythms, require a specific member of a classic nuclear import pathway, Importin α1 (IMPα1). In addition to IMPα1, rhythmic behavior and nuclear expression of PER-TIM require a specific nuclear pore protein, Nup153, and Ran-GTPase. IMPα1 can also drive rapid and efficient nuclear expression of TIM and PER in cultured cells, although the effect on PER is mediated by TIM. Mapping of interaction domains between IMPα1 and TIM/PER suggests that TIM is the primary cargo for the importin machinery. This is supported by attenuated interaction of IMPα1 with TIM carrying a mutation previously shown to prevent nuclear entry of TIM and PER. TIM is detected at the nuclear envelope, and computational modeling suggests that it contains HEAT-ARM repeats typically found in karyopherins, consistent with its role as a co-transporter for PER. These findings suggest that although PER is the major timekeeper of the clock, TIM is the primary target of nuclear import mechanisms. Thus, the circadian clock uses specific components of the importin pathway with a novel twist in that TIM serves a karyopherin-like role for PER.

## Author Summary

In *Drosophila*, circadian rhythms are driven by a negative feedback loop that includes the key regulators, *period* (*per*) and *timeless* (*tim*). To generate this feedback loop, PER and TIM proteins first accumulate in the cytoplasm and then translocate to the nucleus where PER represses transcription. Thus, the nuclear import of PER-TIM proteins is a critical step to separate the phases of activation and repression of mRNA synthesis. In this study, we discovered that a member of the nuclear import machinery, importin α1 is an essential component of this feedback loop. Flies lacking importin α1 (IMPα1) display arrhythmic behavior and cytoplasmic expression of both PER and TIM at all times. In cultured S2 cells, IMPα1 expression directly facilitates nuclear import of TIM, but the effect on PER appears to be indirect. TIM expression is detected at the nuclear envelope and it interacts with other components of the nuclear transport machinery, which we show are also required for nuclear expression of TIM-PER and for behavioral rhythms. Our results thus suggest that TIM functions to link PER to the nuclear import machinery through IMPα1. Altogether, this study provides the mechanistic basis of a crucial step in the circadian clock mechanism.

## Introduction

The mechanisms that generate a circadian (~24 h) clock within organisms have been a subject of investigation for many years. In *Drosophila*, the core clock mechanism consists of a negative feedback loop comprised of the *period* (*per*) and *timeless* (*tim*) genes [1]. Transcription of the *per* and *tim* genes is initiated during mid-day by the transcription factors CLOCK (CLK) and CYCLE (CYC), while the two proteins, PER and TIM, accumulate in the early night and translocate into the nucleus several hours later, to suppress the activity of CLK and CYC. Degradation of TIM in the early morning promotes progressive phosphorylation and ultimately degradation of PER, which releases the repression activity in the nucleus and allows re-initiation of *per* and *tim* transcription. Delayed nuclear entry of the PER-TIM proteins, relative to their first appearance, is essential to generating the clock as it separates the phase of mRNA synthesis from transcriptional repression [2]. Also, precise timing of nuclear entry appears to be critical for generating a clock that maintains accurate period [3–5]. In fact, the timing of nuclear entry and duration of PER and TIM localization in the nucleus are likely among the most critical determinants of circadian period.

For the reasons noted above, the mechanisms underlying the nuclear entry of PER and TIM have been of great interest. The two proteins are believed to regulate each other's nuclear entry, but this has been challenged in many studies conducted both *in vitro* and *in vivo* [6–9]. Analysis of this issue in flies has been complicated by the fact that PER is unstable in the absence of TIM [10,11] making it difficult to assess its localization. In addition, although TIM is stable, and cytoplasmic in *per*-null mutants, it actually shuttles in and out of the nucleus, suggesting that PER is required for its nuclear retention rather than its localization [12]. Overall, the mechanisms that drive nuclear entry of PER-TIM remain unclear although this event is clearly very important in the clock mechanism.

The nuclear transport of proteins is fundamental for regulating cell biogenesis, physiological homeostasis, development and disease [13]. Studies have established a classical nuclear import pathway in which a macromolecule/cargo (greater than 40kDa) containing a nuclear localization signal (NLS) binds to importin α- IMPα [14,15], which in turn binds to importin β- IMPβ, and this ternary complex targets the nuclear pore complex (NPC) proteins to facilitate nuclear translocation of the cargo [14]. After translocation into the nucleus, the binding of nuclear Ran-GTP to IMPβ dissociates the trimeric complex (cargo-IMPα-IMPβ) and the free IMPα and β are recycled back to the cytoplasm by the exportin CAS and Ran respectively [16,17]. This classical pathway is sometimes modified; for instance, nuclear import can be mediated by IMPβ proteins alone (one or more at the same time) [18,19], IMPα has also been shown to carry the cargo protein into the nucleus independent of IMPβ [20], and there are also examples of nuclear translocation through the NPC independent of the involvement of either alpha or beta importins [21]. The *Drosophila* genome encodes a full complement of importins, including at least four importin α homologs (*importin α1*, *α2*, *α3*, and *α4*), as well a Ran-GTPase ortholog [13,14].

Despite what we know about the nuclear import process, none of the classical import factors has been examined for its role in the nuclear entry of PER or TIM. As both PER and TIM qualify as macromolecules (> 40kDa) and each contains at least one NLS that is functional in cells [6,22,23], we speculated that the importin system would be involved in their nuclear localization. We report here that the nuclear localization of PER and TIM in clock cells, and thereby behavioral rhythms in *Drosophila*, depend upon a specific alpha-importin: IMPα1. We also demonstrate roles for other components of the nuclear import machinery—Ran and a nuclear pore protein, NUP153—in the localization of PER-TIM. Based upon structure-function analysis of the IMPα1-TIM interactions, we suggest that TIM is the primary cargo of the importin system, and it acts in a karyopherin-like capacity to co-transport PER. We also identify the molecular basis of a previously identified *tim* mutant as an impaired interaction between TIM and IMPα1.

## Results

### A specific Importin α is required for free-running locomotor rhythms

**Figure 1:**
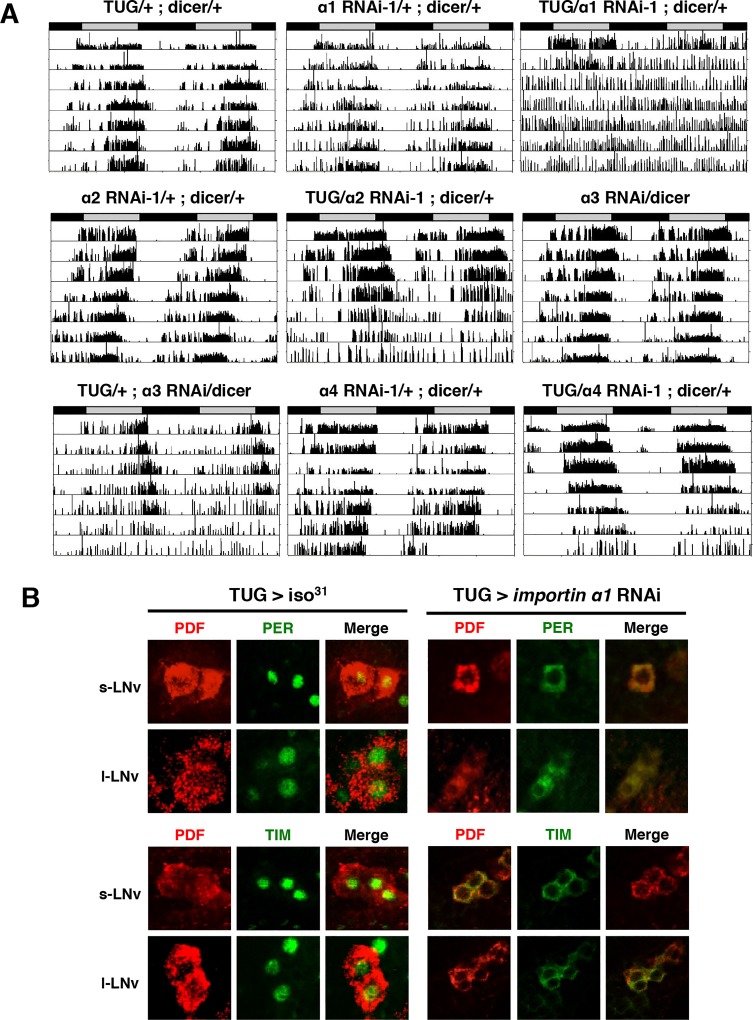
Downregulation of IMPα1 in clock neurons disrupts rest:activity rhythms and nuclear translocation of clock proteins, PER and TIM. (A) IMPα1 knockdown leads to arrhythmia in constant darkness (DD). Genotypes are indicated on the top of each panel. The gray and black bars indicate subjective day and night, respectively. Representative activity records are shown. (B) Downregulation of IMPα1 impairs nuclear translocation of clock proteins, PER and TIM. Control flies and IMPα1 knockdown flies were collected at ZT0 and brains were dissected and stained with antibodies to PER or TIM (green) and PDF (red).

To determine if any member of the importin α family is required for the nuclear entry of PER/TIM, we used the UAS/GAL4 system to knock down the expression of each importin α via RNAi in clock cells. We obtained UAS-importin α RNAi lines from the Vienna-based *Drosophila* stock center (VDRC) or the Bloomington stock center, and crossed each one to *Pdf*-GAL4 and *tim*-UAS-GAL4 (TUG) to achieve knockdown in central clock neurons, the ventral lateral neurons (LNvs), and all clock cells, respectively. Dicer was also coexpressed to increase the efficacy of RNAi. We assayed the locomotor rhythms of these flies under continuous dark conditions (DD) and found that knockdown of importin α1 in PDF-positive LNvs resulted in weak rhythms, as determined through fast fourier transform (FFT) analysis (Table 1). Use of stronger drivers (TUG and *tim*-GAL4) to express importin α1 RNAi in all clock cells rendered all flies arrhythmic in DD (Table 1 and Fig. 1A). We used two independent RNAi lines for importin α1, one from VDRC (α1 RNAi-1) and the other from the Bloomington stock center (α1 RNAi-2). α1 RNAi-1 expressed by TUG produced arrhythmic behavior, which was accompanied by lower mRNA levels of importin α1 (~ 64%). TUG driven α1 RNAi-2 lines did not show a behavioral phenotype, which was consistent with lack of an effect on importin α1 mRNA (S1 Fig.).

**Table 1:** Effects of downregulating importin α genes on free-running circadian locomotor rhythms.

Genotype	n	Rhythmic Flies (%)^a^	Period (hr) ± SEM	Power (FFT) ± SEM
α1 RNAi-1/+; dicer/+	16	100	23.36 ± 0.07	0.087 ± 0.012
α1 RNAi-2/dicer	16	100	23.06 ± 0.06	0.151 ± 0.009
α2 RNAi-1/+; dicer/+	16	100	23.22 ± 0.04	0.111 ± 0.011
α2 RNAi-2/dicer	16	100	23.46 ± 0.12	0.149 ± 0.014
α3 RNAi-1/dicer	16	100	23.80 ± 0.06	0.059 ± 0.008
α4 RNAi-1/+;dicer	16	100	23.62 ± 0.05	0.075 ± 0.009
*Pdf*-GAL4/+; dicer/+	12	100	23.77 ± 0.06	0.056 ± 0.010
*Pdf*-GAL4/α1 RNAi-1; dicer/+	19	84.2	23.98 ± 0.01	0.027 ± 0.003^b^
*Pdf*-GAL4/+; dicer/α1 RNAi-2	14	100	23.68 ± 0.05	0.075 ± 0.006
*Pdf*-GAL4/α2 RNAi-1; dicer/+	15	93.3	23.86 ± 0.04	0.071 ± 0.010^c^
*Pdf*-GAL4/+; dicer/α2 RNAi-2	15	100	24.66 ± 0.07	0.071 ± 0.010
*Pdf*-GAL4/+; dicer/α3 RNAi	16	100	24.23 ± 0.12	0.090 ± 0.008
*Pdf*-GAL4/α4 RNAi-1; dicer/+	15	100	24.04 ± 0.09	0.071 ± 0.007
*tim*-GAL4/+; dicer/+	15	100	23.85 ± 0.04	0.056 ± 0.008
*tim*-GAL4/α1 RNAi-1; dicer/+	14	0	-	-
*tim*-GAL4/+; dicer/α1 RNAi-2	16	100	23.72 ± 0.02	0.107 ± 0.010
*tim*-GAL4/α2 RNAi-1; dicer/+	15	100	24.31 ± 0.10	0.085 ± 0.008
*tim*-GAL4/+; dicer/α2 RNAi-2	16	100	25.17 ± 0.06	0.080 ± 0.009
*tim*-GAL4/+; dicer/α3 RNAi	lethal	-	-	-
TUG/+; dicer/+	15	100	23.68 ± 0.04	0.082 ± 0.011
TUG/α1 RNAi-1; dicer/+	15	0	-	-
TUG/+; dicer/α1 RNAi-2	16	100	23.43 ± 0.06	0.080 ± 0.008
TUG/α2 RNAi-1; dicer/+	15	86.7	24.43 ± 0.09	0.081 ± 0.010^c^
TUG/+; dicer/α2 RNAi-2	16	100	24.92 ± 0.07	0.081 ± 0.009
TUG/+; dicer/α3 RNAi	14	71.4	23.96 ± 0.09	0.027 ± 0.007^c,d^
TUG/α4 RNAi-1; dicer/+	16	100	24.28 ± 0.13	0.139 ± 0.012

^a^ Flies with FFT value > 0.01 are counted as a rhythmic.

^b^ P < 0.005 compared to both α1 RNAi-1/+; dicer/+ and Pdf-GAL4/+; dicer/+ controls, by Student's t-test.

^c^ These flies showed arrhythmicity or weak rhythmicity 4–5 days after transferring to DD. Periods and FFTs during first 4–5 days in DD are shown.

^d^ P < 0.01 compared to both α3 RNAi-1/dicer and TUG/+; dicer/+ controls, by Student's t-test

We found that *Pdf*-GAL4- and TUG-driven importin α2 RNAi also produced arrhythmia or weak rhythmicity, although only after 4–5 days in DD (Fig. 1A). In addition, knockdown of importin α3 by TUG in clock cells resulted in arrhythmia in 50% of the flies after 5 days in DD, and knockdown of importin α3 with a stronger *tim*-GAL4 driver induced lethality (Table 1). Overall, the behavioral phenotype produced by knocking down these other two importins was weaker than that seen for importin α1, even though mRNA levels of the relevant importin were reduced in the flies tested (S1 Fig.). Knockdown of importin α4 knockdown did not produce any behavioral phenotype.

### Knockdown of Importin α1 in clock cells affects the nuclear entry of PER and TIM

To determine whether the arrhythmic behavioral phenotype of importin α1 knockdown flies was caused by blocked nuclear entry of PER and TIM, we examined PER/TIM protein localization in adult LNvs. In control flies, PER and TIM were nuclear at zeitgeber time 0 (ZT0) (ZT0 = lights on and ZT12 = lights off), as has been reported in several previous studies [24–26]. However, both proteins were cytoplasmic at ZT0 in TUG driven α1 RNAi-1 flies (Fig. 1B). We did not observe any anatomical defects in the PDF+ cell bodies or in the PDF projections of LNvs in these flies, suggesting that the observed behavioral and molecular phenotype is not due to gross developmental defects (S2 Fig.).

As mentioned above, flies in which expression of importin α2 or α3 was reduced in adult LNvs or in all clock neurons were also arrhythmic 4–5 days after transferring to DD. To investigate whether importins α2 or α3 also play a role in the nuclear entry of clock proteins, we immunostained whole-mount brains of α2 and α3 knockdown flies using a PER antibody at ZT1. In both fly lines, PER was expressed in the nucleus at ZT1 (S2 Fig.), indicating that neither importin α2 nor α3 is required for the nuclear translocation of PER in LNvs. However, the distal layer of the optic medulla, including the PDF projections of large LNvs into the medulla, was impaired in these flies, suggesting that importin α2 and α3 contribute to the development of the LNvs. Thus, the arrhythmic phenotype observed after a few days in DD in flies with reduced importin α2 and α3 may derive from developmental problems. We infer that only importin α1 affects the nuclear entry of PER and TIM in central clock cells.

### The importin α1 deletion mutant, Df(3L)α1S1, recapitulates the phenotypes of RNAi flies

**Figure 2:**
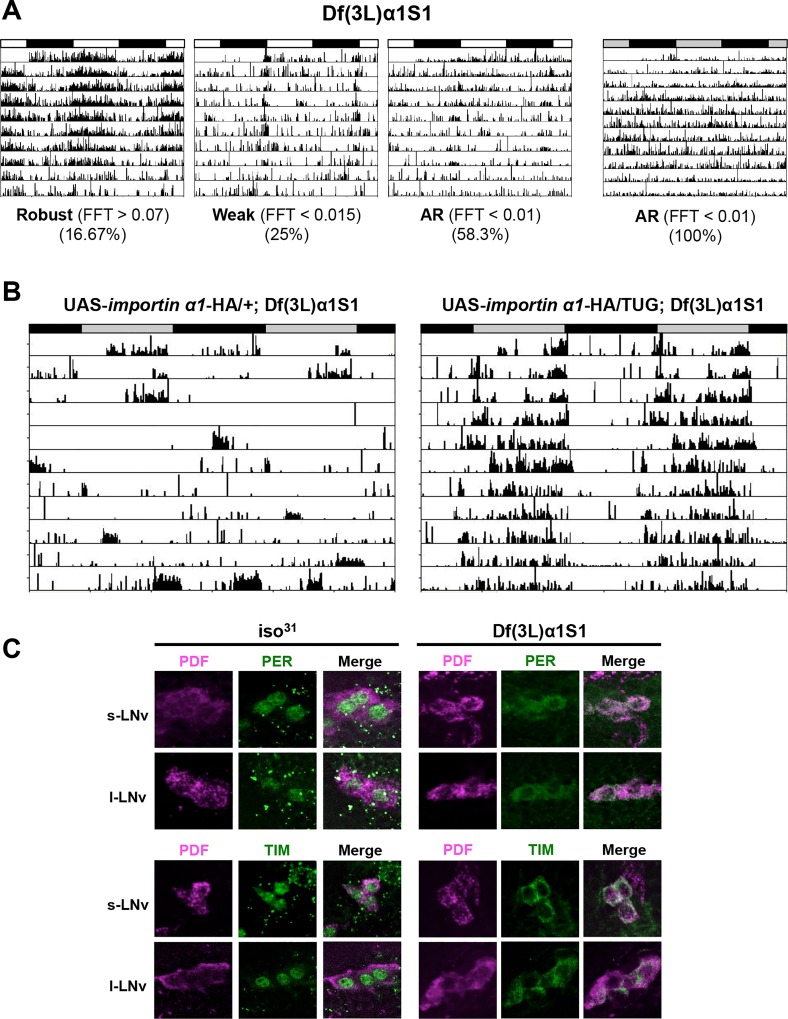
An IMPα1 deletion mutant, Df(3L)α1S1, recapitulates the phenotypes of IMPα1 knockdown flies. (A) Complete loss of IMPα1 results in weak rhythms in LD and complete loss of rhythms in DD. Flies were categorized as arrhythmic (AR), or having weak or robust rhythms based on FFT values as indicated beneath the panels. The white and black bars indicate day and night in LD cycles and the gray and black bars indicate subjective day and night in DD. (B) Activity records of individual UAS-*importin α1*-HA/+; Df(3L)α1S1 (left) and UAS-*importin α1*-HA/TUG; Df(3L)α1S1 (right) in DD. Expression of IMPα1 in all clock neurons using TUG in Df(3L)α1S1 flies rescues behavioral rhythms in DD. Rescue experiments were also conducted using *pdf*-Gal4 (Table 2). (C) Blocked nuclear entry of PER and TIM in LNvs of Df(3L)α1S1 flies. Brains were dissected from wild-type flies (iso^31^) and Df(3L)α1S1 flies and then stained for PER or TIM (green) and PDF (purple) on the 3^rd^ day of LD at ZT0.

Given the limitations with RNAi technology, such as nonspecific and off-target effects [27], we sought to verify the results of RNAi knockdown using a complete loss-of-function allele of importin α1- Df(3L)α1S1. This allele consists of a deletion, which removes the entire importin α1 gene and several other genes, and is homozygous viable [28]. Consistent with the phenotype obtained by knocking down importin α1, Df(3L)α1S1 flies were arrhythmic in DD. These flies also showed reduced rhythmicity in the presence of light:dark cycles, with ~60% of flies scored as arrhythmic (Table 2 and Fig. 2A). As the deletion in Df(3L)α1S1 flies includes multiple genes besides importin α1, we sought to determine whether the mutant phenotype derived exclusively from the loss of importin α1. We tagged a cDNA encoding wild type importin α1 with an HA epitope and cloned it under control of a UAS sequence and then expressed in the Df(3L)α1S1 background using the GAL4 system. In DD, expression of importin α1 in clock neurons using TUG restored rhythmicity in 84% of Df(3L)α1S1 flies (Table 2 and Fig. 2B). We were also able to rescue rhythmicity in 70% of mutant flies using the *Pdf*-GAL4 driver, suggesting that expression of importin α1 is predominantly required in LNvs for free running locomotor rhythms (Table 2). These results demonstrate that importin α1 is essential for maintaining rhythms in DD and LD.

**Table 2:** Free-running circadian locomotor rhythms in the importin α deletion mutant, Df(3L)α1S1, and rescue lines.

	Genotype	n	Rhythmic Flies (%)	Period (hr) ± SEM	Power (FFT) ± SEM
LD	iso^31^	16	100	23.68 ± 0.04	0.104 ± 0.007
	Df(3L)α1S1	14	41.7	23.97 ± 0.04	0.038 ± 0.002
DD	iso^31^	15	100	23.96 ± 0.07	0.089 ± 0.006
	Df(3L)α1S1	91	0	-	-
	UAS-*importin α1*-HA/TUG; Df(3L)α1S1	56	83.9	23.78 ± 0.05	0.062 ± 0.005
	UAS-*importin α1*-HA/*Pdf*-GAL4; Df(3L)α1S1	44	70.5	24.48 ± 0.13	0.059 ± 0.009

To investigate whether nuclear translocation of PER/TIM was affected in Df(3L)α1S1 mutant flies, we performed immunostaining. At ZT0, PER and TIM were completely cytoplasmic in mutant flies while they were nuclear in wild-type flies, recapitulating the result obtained with importin α1 RNAi flies (TUG > importin α1 RNAi-1) (Fig. 2C). Over a 24 hour cycle, as expected, the localization and expression of PER was rhythmic in wild type flies, such that it was nuclear at ZT0 and ZT6 (with lower levels at ZT6 than ZT0), cytoplasmic at ZT18 and practically undetectable at ZT12 (S3A Fig.). In mutant flies, PER was cytoplasmic whenever detected, but expression was undetectable at ZT12, indicating that its levels are regulated in the cytoplasm. Under DD conditions, in wild type flies, the pattern of localization and expression of PER were similar to those in LD. However, in mutants PER was cytoplasmic at all times and levels did not cycle (S3B Fig.). Likewise, the TIM expression pattern in wild-type flies (nuclear at ZT0, expressed at very low levels at ZT6 and 12 and cytoplasmic at ZT18) was similar in LD and DD except that expression was slightly higher at CT12 than ZT12 (S3C/D Fig.). In mutant flies, TIM was cytoplasmic at ZT0 and ZT18 and undetectable at ZT6 and ZT12, presumably due to its degradation by light. However, in DD TIM was detected in the cytoplasm at all times (S3D Fig.). These data are also supported by studies of temporal patterns of PER and TIM protein accumulation in fly heads as discussed below.

The deletion mutant of importin α1, Df(3L)α1S1, confirmed that importin α1 is important for mediating the nuclear entry of clock proteins, PER and TIM, and thus maintaining behavioral rhythms. We also determined if IMPα1 produces an over-expression phenotype in *impα1* heterozygotes (Df(3L)α1S1/+), which show wild type behavior, or in period-altering *per* mutants, *per* ^*L*^ and *per* ^*S*^. The *per* ^*L*^ mutation, which increases circadian period, was of particular interest as it is associated with delayed nuclear entry of PER and TIM. However, IMPα1 expression did not affect rhythms of “wild type” flies (S4A Fig.) or the periodicity of *per* mutants (S4B/C Fig.). These data suggest that while IMPα1 is required for nuclear expression of PER and TIM, it is typically not a limiting factor in flies.

### Molecular oscillations of PER and TIM are abrogated in Df(3L)α1S1 flies

**Figure 3:**
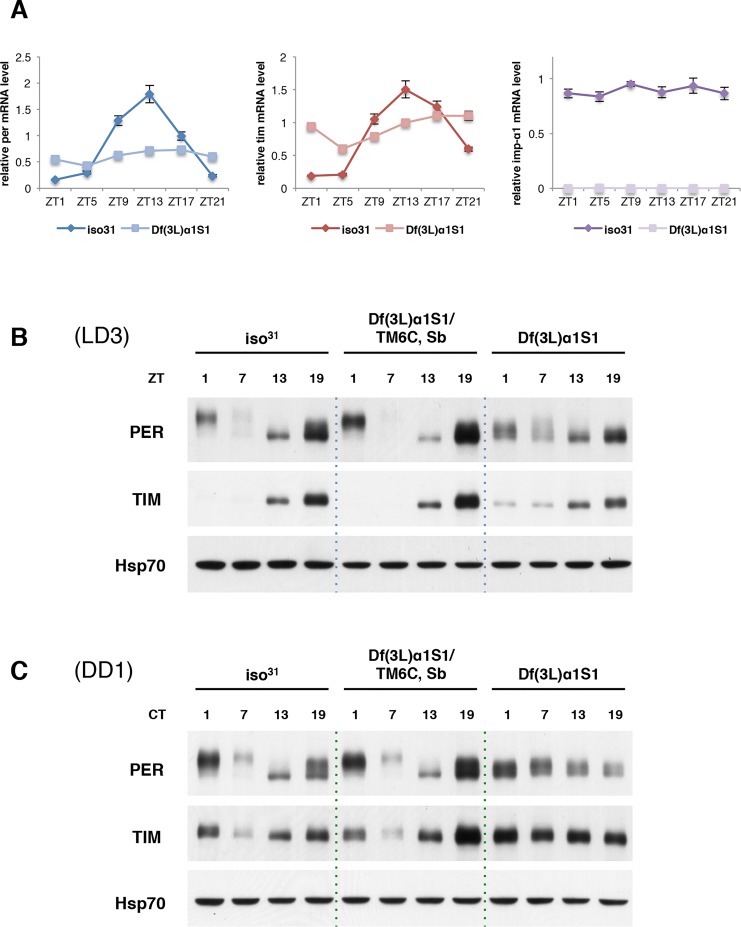
Molecular oscillations of PER and TIM are dampened in Df(3L)α1S1 flies. (A) Quantitive PCR (qPCR) reveals that *per* and *tim* mRNA oscillations are blunted in Df(3L)α1S1 flies relative to those in wild-type flies. *importin α1* mRNA levels do not cycle. *actin* was used as an internal control to normalize transcript levels. The quantification curves in each panel were plotted as average ± standard error of the mean (SEM) of three independent experiments. (B and C) Western blots of adult fly heads of wild-type (iso^31^), heterozygous, and homozygous Df(3L)α1S1 flies were probed for PER and TIM. Flies of the indicated genotypes were collected (B) at different zeitgeber times (ZT) on the 3^rd^ day of LD (LD3) and (C) at different circadian times (CT) on the 1^st^ day of DD (DD1). The blots were also probed with antibody to Hsp70 as a loading control. Similar results were obtained in two or three independent experiments. The quantification of TIM expression levels is shown in S5 Fig.

From the late night to early morning, nuclear PER and TIM inhibit their own transcription, resulting in low levels of both mRNAs. As nuclear TIM is degraded in response to light at daybreak and then PER is hyperphosphorylated and also degraded, repression activity is released and levels of *per* and *tim* mRNA rise [1,2]. We examined whether blocked nuclear entry of PER and TIM in Df(3L)α1S1 mutant flies affects cycling of their mRNAs. We expected that RNA levels of *per* and *tim* in mutant flies would not cycle and levels would be higher than those of wild-type flies at all times over the course of a day due to reduced repression. Consistent with our expectation, mRNA levels did not cycle. However, we noticed higher levels of *per* and *tim* mRNA, relative to wild type, only during the normal trough of mRNA expression, which corresponds to nuclear expression of TIM and PER (ZT21-ZT1) (Fig. 3A). At other times, RNA levels were lower in the mutant. It is possible that release of transcriptional repression in wild type flies leads to more efficient gene expression than constitutive transcription, which could account for this effect. We also tested whether mRNA levels of *importin α1* are expressed cyclically. Quantitative PCR (qPCR) revealed that *importin α1* transcript levels do not cycle.

We then assayed the expression of PER and TIM proteins in whole heads of Df(3L)α1S1 mutant flies. Western blots of fly head extracts from different time points indicated 24 hour oscillations of PER and TIM protein levels in LD. However, the amplitude of the oscillation was blunted relative to wild type (Fig. 3B). As reported previously [25], hyperphosphorylated forms of PER, detected as low mobility forms on the gel, are observed predominantly at times of nuclear expression e.g. at ZT1. These are the forms that are subsequently targeted for degradation. PER in mutant flies was less phosphorylated at ZT1 and more stable at ZT7 than in wild-type (Fig. 3B). In DD, it appeared that PER stability, but not its phosphorylation, still cycled in mutant flies, albeit with an aberrant and variable phase (Fig. 3C). Thus, the stability of PER may fluctuate in the cytoplasm. It is also possible that the translocation of PER is regulated in an importin α1-independent manner in some cells that contribute to the signal on whole head western blots. Based upon the lack of cyclic phosphorylation, however, we favor the explanation that PER remains in the cytoplasm.

As for PER, TIM cycling was dampened in importin α1 mutants. TIM levels were reduced at daybreak in mutant flies, suggesting that light can degrade cytoplasmic TIM; indeed, the weak cycling seen for PER could be driven by light-induced cycling of TIM, as TIM stabilizes PER [10,11,29]. However, some TIM remained in mutant flies at ZT1 while TIM in wild type flies was completely gone (Fig. 3B and S5A Fig.). It is possible that cytoplasmic TIM is less sensitive to light-driven degradation. On the other hand, as TIM degradation around dawn is also regulated by circadian clock mechanisms, which persist in constant darkness [30], we speculate that clock-dependent degradation of TIM requires nuclear expression. Consistent with this idea, TIM cycling was completely abolished in DD (Fig. 3C and S5B Fig.).

### IMPα1 affects PER/TIM nuclear translocation in S2 cells

**Figure 4:**
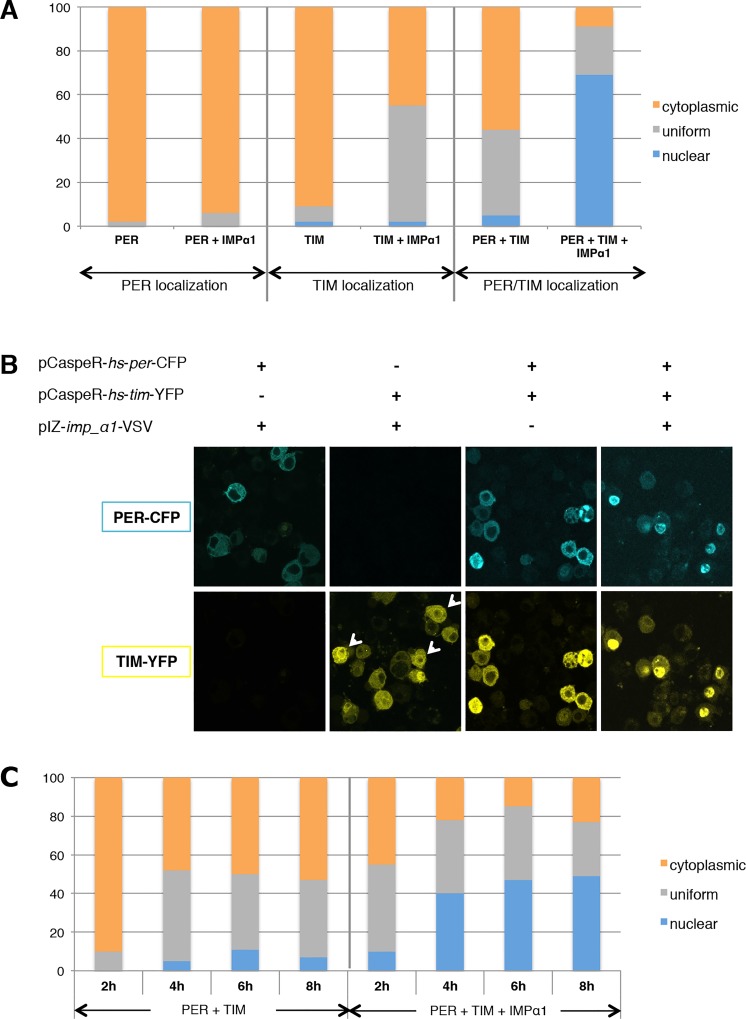
IMPα1 overexpression increases PER/TIM nuclear translocation in S2 cells. (A and B) S2 cells were transiently transfected with pCaspeR-*per*-*cfp*, pCaspeR-*tim*-*yfp*, and pIZ-*importin α1*-VSV as indicated. (A) Cells were fixed 8 hrs after heat shock induction and then scored as nuclear (blue), cytoplasmic (orange), and uniform (both nuclear and cytoplasmic; gray). At least 100 cells were counted for each condition in two independent experiments. (B) Examples of each condition. The white arrowhead indicates nuclear envelope association of TIM (see also Results and S7B Fig.). (C) S2 cells were transfected as in Fig. 4A and then fixed every two hours after heat shock induction. S2 cells were counted as shown above.

Given the importance of IMPα1 in the nuclear translocation of PER/TIM in clock cells, we sought to determine if its effect on the subcellular localization of clock proteins could be recapitulated in S2 cells. Both *per* and *tim* were tagged with CFP and YFP respectively, and their expression was induced by heat-shock as previously described [9,25]. Previous studies have shown that either PER or TIM alone is cytoplasmic when transfected into S2 cells, but each shows more nuclear expression when the other protein is co-expressed [12,22,25]. However, even under these conditions, neither protein becomes completely nuclear in the S2 cells we use [25]. As these cells likely express very low levels of IMPα1, we wondered if it was the missing component, and so added IMPα1 in these experiments. The addition of IMPα1 had no significant effect on PER localization by itself, but it increased uniform (nuclear and cytoplasmic) expression of TIM up to almost 50% (Fig. 4A/B). As previously reported [22,25], co-expression of PER and TIM also increased nuclear and uniform expression of both proteins (40–50%). Interestingly, co-transfection of all three proteins (PER, TIM and IMPα1) resulted in strong and specific nuclear localization of PER/TIM in ~70% of cells, with most other cells showing uniform expression (Fig. 4A/B). These results suggested that IMPα1 primarily facilitates nuclear entry of TIM in S2 cells.

Previous studies have found a delay in the nuclear translocation of PER/TIM following induction of their expression in S2 cells. Thus, they remain in the cytoplasm for 5–6 hours and then transfer into the nucleus [9,22]. This delay is reminiscent of the delay in nuclear entry observed in clock cells, a delay that is thought to be critical for clock function [24]. To investigate whether IMPα1 might be a rate-limiting factor that causes the delay in nuclear transport of PER/TIM in S2 cells, we followed subcellular localization every 2hr after heat-shock induction of both proteins. When PER and TIM were expressed together without IMPα1, they were mainly cytoplasmic 2hr after induction and showed increased uniform and nuclear expression 4hr after induction. However, increased nuclear and uniform expression of PER and TIM was detected even 2hr after induction when IMPα1 was co-expressed with PER/TIM (Fig. 4C). Thus, the delay in S2 cells may typically result from low expression levels of endogenous IMPα1.

As S2 cells vary from batch to batch, we also examined a different sub-culture that shows higher nuclear expression (~85%) of co-expressed PER and TIM [9]. Interestingly, these cells express detectable levels of IMPα1 (S6A Fig.), which may account for the increased nuclear expression. To determine if this was the case, we down-regulated IMPα1 via RNAi, and found that it confined the expression of PER and TIM to the cytoplasm; however, it did not affect nuclear expression of dCLK in S2 cells (S6A/B Fig.). Together these data support the idea that IMPα1 is specifically required for the nuclear translocation of PER/TIM in S2 cells.

### The nucleoporin, NUP153, and Ran-GTP are also required for nuclear entry of PER and TIM

**Figure 5:**
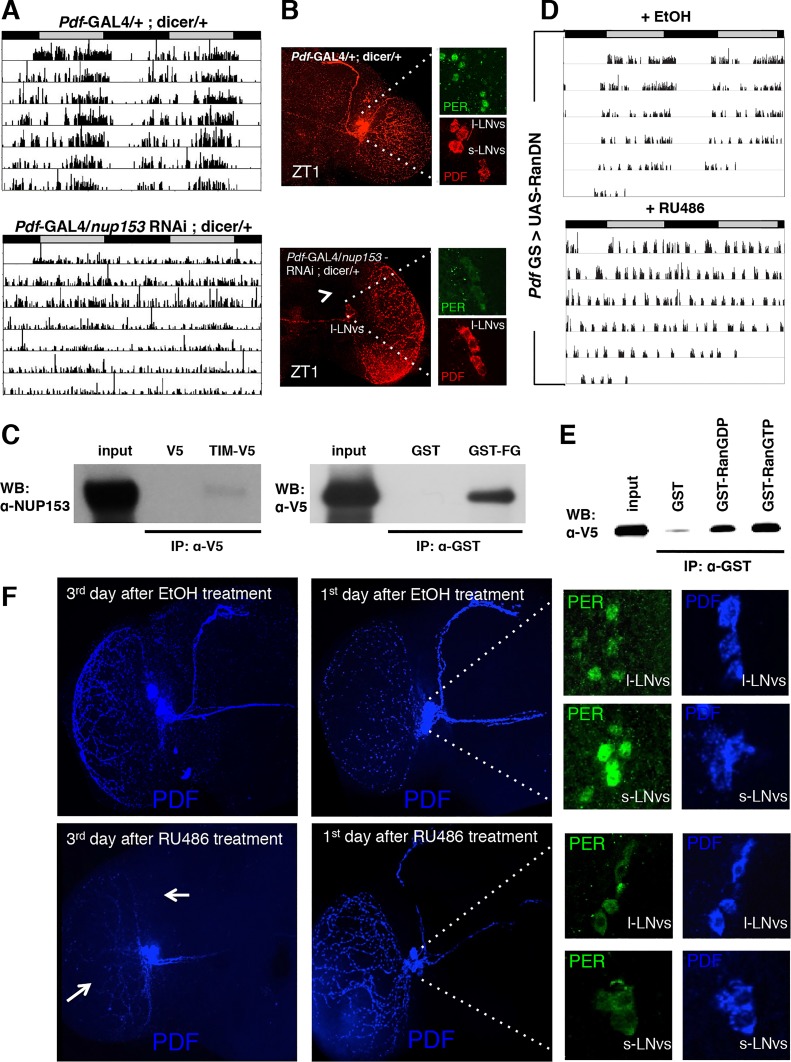
NUP153 and RAN are required for circadian rhythms and interact with TIM. (A) Knockdown of Nup153 disrupts rest:activity rhythms. Representative activity records of individual pdf-Gal4/+; dicer/+ (left) and pdf-Gal4/*nup153* RNAi; dicer/+ (right) flies are shown. (B) PDF expression in nup153 knockdown flies was detected in l-LNvs, but not in the s-LNvs. In nup153 knockdown flies (right), the s-LNv dorsal tract was impaired as indicated by the white arrow. Low, but cytoplasmic expression of PER (green) was detected in l-LNvs at ZT1. In control flies (left), PER (green) was nuclear in both s- and l-LNvs at ZT1. (C) S2 cells were transfected with pIZ-V5 or pIZ-*tim*-V5 constructs. After 60 hours, cell lysates were immunoprecipitated with an anti-V5 antibody and western blots were probed with an anti-NUP153 antibody (left). GST pulldown assays were also conducted to test for an interaction between the FG repeat of NUP153 (GST-FG) and TIM (right). Cell extracts from S2 cells transfected with pIZ-*tim*-V5 were incubated with purified recombinant GST-FG or a GST control. Proteins pulled down by GST were analyzed with an anti-V5 antibody (that recognizes TIM-V5). Similar results were obtained in three independent experiments. (D) Expression of a dominant negative form of Ran (RanDN) in PDF^+^ cells during adulthoods leads to arrhythmia in DD. RanDN was expressed under control of an RU486-inducible *Pdf*-GS driver. Flies were fed either 500 mM RU486 or ethanol (EtOH, vehicle control) from the time of entrainment. (E) GST pulldown experiments detect interactions between TIM expressed in S2 cells and purified GST-RAN fusion proteins. RAN was tested in GDP-bound (RanGDP) and GTP-bound (RanGTP) forms. GST alone served as a control. The bound protein was analyzed with an anti-V5 antibody. In five independent experiments we have confirmed that TIM shows slight preference (1.64 ± 0.18) for Ran GTP over Ran GDP. (F) Expression of RanDN in central clock cells leads to severe morphological defects (white arrow) on the 3^rd^ day after RU486 treatment. On the 1^st^ day after RU486 treatment, morphological effects were not visible and PER (green) was cytoplasmic in both l-LNvs and s-LNvs at ZT1.

Importins typically drive nuclear transport of cargoes in concert with a number of other proteins, including nuclear envelope proteins (nucleoporins). Interestingly, we have frequently noticed nuclear envelope association of TIM in LNvs of adult flies at ZT20 (S7A Fig.). This expression of TIM at the nuclear rim was also observed for TIM transfected in S2 cells (Fig. 4B and S7B Fig.). To investigate whether nucleoporins (NUPs) are part of the circadian mechanism, we knocked several NUPs (NUP214, 88, 153, 154 and Megator) down in all clock cells using TUG with dicer and assayed rest:activity behavior. None of these showed significant change in circadian behavior compared to control flies. However, knockdown of NUP153 resulted in lethality. To circumvent the lethality, we restricted the expression of *nup153* RNAi to adult clock neurons by coupling the TUG driver with *tublin-GAL80* ^*ts*^. We found that these flies also died 3–4 days after being moved to 29°C. However, they were arrhythmic before they died (S7C Fig.). Use of the weaker *Pdf*-GAL4 driver (along with dicer) to downregulate NUP153 expression dampened rhythmicity of flies without causing any lethality (Fig. 5A and S1 Table). As with the importins, we assayed expression of PER in the PDF^+^ cells of flies that had NUP153 knocked down. Knockdown of NUP153 disrupted the s-LNvs, and so we assayed PER in l-LNvs and found that it was cytoplasmic at ZT1, when PER is nuclear in control flies (Fig. 5B). These data indicate that while important for development, NUP153 is also required for the nuclear entry of clock proteins.

Given the nuclear envelope localization of TIM, sought to determine if NUP153 binds TIM. Co-immunoprecipitation assays in S2 cells revealed a specific interaction between V5-tagged TIM and endogenous NUP153, which we confirmed through GST-pull down assays. A GST-NUP153FG fusion protein, in which phenylalanine glycine (FG) repeats of NUP153 were fused to GST and expressed in E. coli, interacted with TIM-V5 expressed in S2 cells (Fig. 5C).

Another key regulator of importin-mediated nuclear transport is the small GTPase Ran, which is predominately GDP-bound in the cytoplasm and GTP-bound in the nucleus. In the nucleus Ran-GTP dissociates the IMPα/IMPβ/cargo complex by binding to IMPβ [17,31]. To examine the role of Ran in flies, we expressed a dominant negative form of Ran (RanDN) in adult PDF^+^ neurons using the inducible *Pdf*GeneSwitch (*Pdf*-GS) driver. RU486-induced expression of RanDN caused arrhythmia under DD conditions (Fig. 5D and S1 Table). To investigate whether this behavioral phenotype was related to altered nuclear translocation of clock proteins, we determined the localization of PER in *Pdf*-GS > UAS-RanDN flies at ZT1 on the 3^rd^ day after RU486 treatment. In these flies PDF expression and the dorsal projection from sLNvs were severely impaired. Also, PER was difficult to detect, presumably due to low expression levels. To minimize these effects of Ran, we assayed the localization of PER at ZT1 on the 1^st^ day after RU486 treatment, as morphological effects were not visible at this time. PER was cytoplasmic at ZT1 in RanDN flies whereas it was nuclear in control flies (Fig. 5F), suggesting that Ran is needed for nuclear translocation of clock proteins. Interestingly, we found that TIM expressed in S2 cells also interacts with GST-Ran and this interaction is enhanced when Ran is in the GTP-bound form (Fig. 5E).

### TIM is primary cargo for IMPα1 and serves to co-transport PER

**Figure 6:**
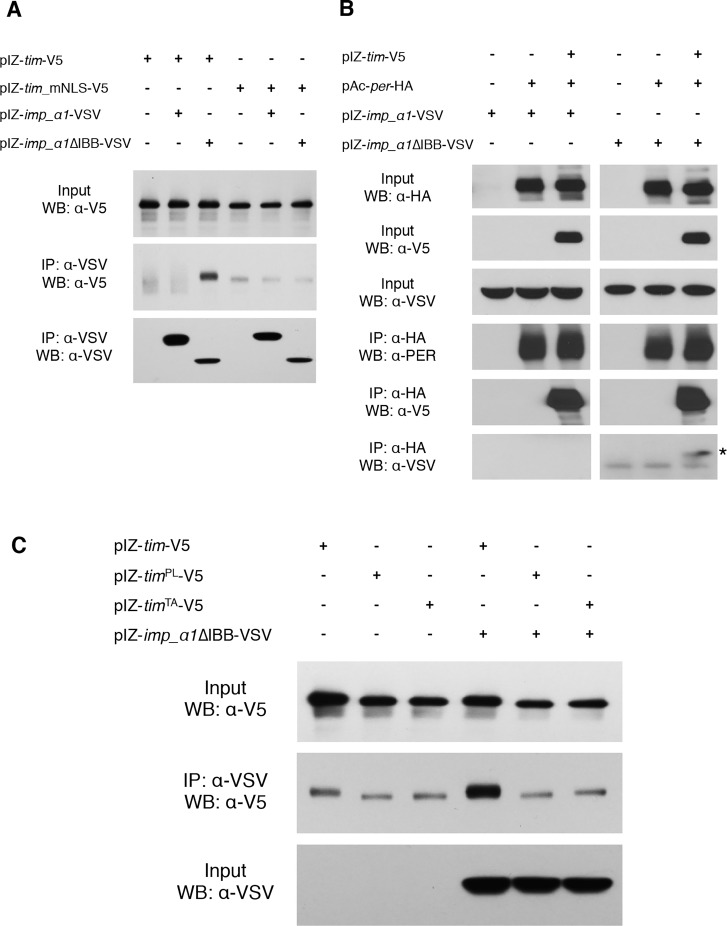
TIM is primary cargo for IMPα1. (A) S2 cells were transiently transfected with pIZ-*tim*-V5 (wt), pIZ-*tim*_mNLS-V5 (*tim* carrying a mutant NLS), pIZ-*imp_α1*-VSV, or pIZ-*imp_α1*∆IBB-VSV as indicated. After 60 hours, cell lysates were immunoprecipitated with an anti-VSV antibody (against IMPα1 or IMPα1IBB) and detected with an anti-V5 antibody. Similar results were obtained in three independent experiments. (B) S2 cells were transiently transfected with pIZ-*tim*-V5, pAc-*per*-HA, pIZ-*imp_α1*-VSV, or pIZ-*imp_α1*∆IBB-VSV as indicated. After 60 hours, cell lysates were immunoprecipitated with an anti-HA antibody (against PER) and detected with an anti-V5 antibody or with an anti-VSV. Similar results were obtained in three independent experiments. IMPα1 co-immunoprecipitated with PER is indicated with an asterisk. (C) S2 cells were transfected with pIZ-*tim*-V5 (wt), pIZ-*tim* ^PL^-V5, or pIZ-*tim* ^TA^-V5 in the presence or absence of pIZ-*imp_α1*∆IBB-VSV as indicated. After 60 hours, cells were subjected to IP using an anti-VSV antibody (against empty vector or IMPα1∆IBB) and detected with an anti-V5 antibody. Similar results were obtained in three independent experiments. The quantification of interaction between IMPα1 and TIM^WT/PL/TA^ is shown in S9 Fig.

Our cell culture data indicated that TIM was required for the nuclear localization of PER by IMPα. This could reflect co-transport of two cargoes, or perhaps even an IMPβ like function for TIM. To further test this idea, we sought to determine whether TIM interacts with IMPα1 in a manner that would be predicted for an IMPβ. IMPα proteins bind IMPβ through the Importin-β-binding (IBB) domain and this interaction modulates the binding of cargo to IMPα [32]. Specifically, the IBB domain folds over and interacts with the NLS-binding site on IMPα and so it competes with cargo for binding to this site [32]. Cargo binding is facilitated when the IBB domain binds IMPβ and releases the NLS-binding site [33,34]. We hypothesized that if TIM functioned as an IMPβ it would not bind to an IMPα1 that lacked the IBB domain. Contrary to our expectations, V5-tagged wild-type TIM showed strong binding to VSV-tagged IMPα1∆IBB whereas it did not bind to wild-type IMPα1 (Fig. 6A). Previous work has shown that the binding affinity of IMPα for cargo increases when the IBB domain is mutated [35]. This result suggests that TIM is cargo for IMPα1 rather than an IMPβ- like molecule.

The idea that TIM is classical cargo for importins is also supported by the finding that mutation of the TIM NLS lengthens the circadian period in flies (~30hr), significantly delays nuclear accumulation of both TIM and wild-type PER in S2 cells, and affects molecular oscillations of PER and TIM in the same manner as the importin α1 mutation [23]. Also, previous work indicated that the nuclear entry of mCRY2, a mammalian clock component that acts as a PER partner, is mediated by the importin α/β system through a bipartite NLS [36]. To determine if the interaction between TIM and IMPα1∆IBB was dependent on the TIM NLS, we mutagenized basic residues of the NLS to alanines. TIM carrying this mutated NLS did not bind IMPα1∆IBB (Fig. 6A), suggesting, as expected for a typical cargo protein, that the NLS mediates the interaction with IMPα1. In addition, we found that mutation of the TIM NLS prevented nuclear expression of TIM and PER co-expressed in S2 cells with IMPα1 (S8A Fig.).

We also assayed interactions between PER and IMPα1 by immunoprecipitating PER with an anti-HA antibody (against PER) and assaying the pellets for expression of IMPα1. Wild type IMPα1 was not pulled down with PER regardless of the presence of TIM. IMPα1∆IBB was pulled down by PER, but only when TIM was co-expressed, suggesting that TIM serves as a bridge between PER and IMPα1∆IBB (Fig. 6B). Based upon sequence analysis, PER has at least two monopartite NLSs: one near the N-terminus (aa 73–77) and the other in the middle of the protein (aa 788–791). However, mutations in these NLSs have little to no effect on the nuclear localization of PER [6]. Chang et al. identified a functional bipartite NLS near the C-terminus between amino acids 813–840. However, mutations in this NLS did not affect the nuclear entry of PER and TIM co-expressed with IMPα1 (S8A Fig.). Thus, TIM is likely the primary target of IMPα1 and its nuclear entry is regulated by the canonical nuclear entry mechanism described above; however, at the same time TIM acts as a carrier for PER.

To assay the interaction between IMPα1 and PER/TIM in flies, we immunoprecipitated HA-tagged IMPα1 from head extracts of flies expressing IMPα1-HA under control of the TUG driver. TIM was pulled down in the early night as well as the late night (S8B Fig.). PER was only detected over background in the late night, but we note that PER levels are also substantially higher at this time.

Although the TIM structure has not been experimentally determined yet, it was previously proposed to belong to an ARM/HEAT protein superfamily [37]. ARM (for Armadillo) and HEAT (for Huntingtin, Elongation factor 3, regulatory subunit A of Protein phosphatase 2A, and Target of rapamycin) repeats are structural units of two (HEAT) or three (ARM) α-helices which form one turn of a superhelix [38]. Intriguingly, karyopherins (both importins and exportins) and even some nuclear pore complex proteins belong to the ARM/HEAT family. Using diverse up-to-date homology modeling programs, we also observed that TIM is weakly homologous to proteins in the ARM/HEAT superfamily or more specifically to karyopherins (S8C Fig.). In addition, TIM shuttles in and out of the nucleus, as do importin molecules [12]. Taken together, these data (requirement of TIM for PER nuclear entry, TIM expression at the nuclear rim, TIM binding with NUP153 and Ran, and homology modeling of TIM) support the idea that TIM serves as a karyopherin-like protein for PER.

### The *tim* ^PL^ mutation affects the interaction between TIM and IMPα1

We sought to determine if the mechanism uncovered here contributes to the cytoplasmic expression of TIM and PER caused by the mutant *tim* ^PL^ allele. This proline 115 to leucine mutation leads to behavioral and molecular phenotypes similar to those observed in IMPα1 mutant flies. In addition, mutation of a nearby threonine residue at 113 (TIM^TA^) recapitulates the cytoplasmic expression of TIM^PL^ in S2 cells and the behavioral/molecular phenotypes of *tim* ^PL^ mutant flies [25]. We assayed interactions of each of these proteins with IMPα1∆IBB. In transfected S2 cells, we observed interactions between wild-type TIM and IMPα1∆IBB, but neither mutant TIM, TIM^PL^ or TIM^TA^, bound IMPα1∆IBB (Fig. 6C and S9 Fig.). Lack of an interaction with IMPα1 likely accounts for the cytoplasmic localization, and thereby arrhythmic behavior, of the *tim* ^PL^ mutant. These data suggest that the interaction with nuclear transport machinery is dependent on the phosphorylation state of TIM. Phosphorylation likely drives a conformational change in TIM, exposing the NLS for interaction with the cargo-binding site on IMPα1.

Together these data indicate that TIM is primary cargo for IMPα1 and it is transported through a classical nuclear entry mechanism, but at the same time, it co-transports PER and thus acts as a karyopherin-like molecule.

## Discussion

**Figure 7:**
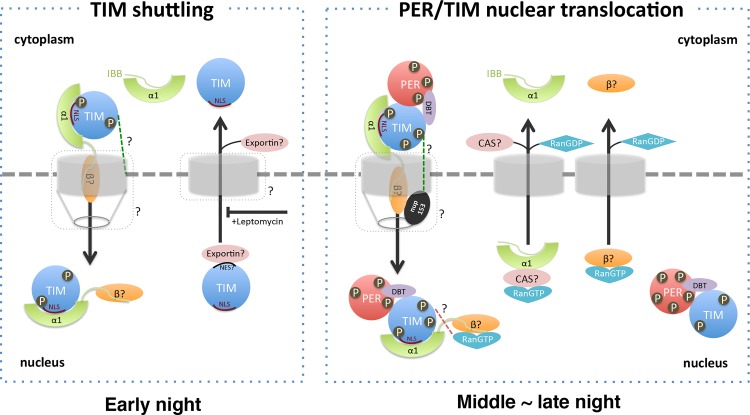
A model for nuclear translocation of Drosophila clock proteins, PER and TIM. Left: In the early night, TIM shuttles in and out the nucleus. TIM is recognized by IMPα1 in the cytoplasm and translocated into the nucleus. The interaction between TIM and IMPα1 is regulated by phosphorylation of TIM. Nuclear entry of the TIM- IMPα1 complex may involve other components like Ran or NUP153 in the nuclear pore complex (green dotted line). In the nucleus, TIM binds to an unknown exportin and is transported back to the cytoplasm, a process that is inhibited by Leptomycin. Right: In the middle to late night, the TIM-IMPα1 complex transports PER into the nucleus, which then leads to the retention of TIM. Note that we cannot exclude the possibility that PER is also transported earlier in the night, but not retained in the nucleus. We speculate that at a specific time, TIM and/or PER is modified by phosphorylation events, which alters the conformation of the PER-TIM-IMPα1 complex and promotes nuclear expression of first PER and then TIM. The PER-TIM-IMPα1 complex binds to IMPβ and passes though the nuclear pore complex containing NUP153. In the nucleus, RanGTP binds to IMPβ, dissociating the complex and releasing the cargo proteins, PER and TIM (a possible direct interaction between TIM and RanGTP is indicated by a red dotted line). After its release from the importin machinery, PER retains TIM in the nucleus. As per the canonical nuclear entry mechanism, IMP α1 and β are likely recycled back to the cytoplasm by exportin Cas and RanGTP, respectively. Question marks indicate unknown processes or components that need to be experimentally validated.

The mechanisms underlying the nuclear translocation of PER and TIM have been debated ever since the *tim* mutation was identified in 1994 [39]. At the time, cytoplasmic localization of a PER-β galactosidase fusion protein in *tim* ^*01*^ mutants and nuclear expression of this fusion protein in wild type was taken as evidence that TIM was required for nuclear expression of PER [29]. Efforts to examine endogenous PER were unsuccessful as PER was found to be very unstable in *tim* ^*01*^ mutants [10,11]. Subsequent cell culture studies indicated that PER and TIM are required for each other's nuclear expression in S2 cells [22], but this result was also questioned by reports indicating that high expression of PER alone represses transcription [6,7,12], and hence enters the nucleus. In addition, studies in *Drosophila* suggested that PER enters the nucleus before TIM [24], and that PER is nuclear in *tim* ^*01*^ mutants when the destabilizing kinase, casein kinase 1ε (known as double-time (DBT) in *Drosophila*), is also removed [8]. However, we found that when PER is stabilized in *tim* ^*01*^ mutants through mutation of a critical destabilizing phosphorylation site (S47), it is cytoplasmic [25,40]. Moreover, we identified a new mutation in *tim*, *tim* ^*PL*^, that allows stable expression of TIM and PER, but prevents their nuclear localization, indicating that TIM is required for nuclear expression of PER [25]. Also, mutating the putative NLS in TIM delays the nuclear entry of TIM and PER [23]. Together, these recent findings supported an important role for TIM in the nuclear expression of PER. Here we identified the mechanisms by which TIM and PER are transported to the nucleus (Fig. 7). We show that TIM is primary and classical cargo for the importin pathway, and it may act in a karyopherin-like capacity to transport PER. We also find that deficits in the interaction with this pathway underlie the phenotype of the *tim* ^*PL*^ mutant.

While some of our data suggested the possibility that TIM serves as an IMPβ for nuclear entry of PER/TIM (Fig. 5), additional tests show that TIM is the primary clock cargo of a specific importin α: IMPα1. In addition, an IMPβ is likely required, indicated by the finding that the IBB domain is required for IMPα1-driven nuclear localization of PER-TIM (S8A Fig.) (note that cargo binding is actually better without the IBB domain).

Although we detected interactions of TIM with NUP153 and Ran proteins using immunoprecipitation (IP) assays, binding of TIM to these proteins may not necessarily be direct. The source of TIM for the immunoprecipitation experiments was transfected protein from S2 cells, which also express many endogenous proteins. Thus, even though we expressed NUP153/RAN in *E*.*coli*, we cannot exclude the possibility that other proteins from S2 cells bridged the interaction. As noted above, both NUP153 knockdown flies and RanDN flies showed morphological defects in clock cells as well as low expression of PER, suggesting that NUP153 and RAN also regulate clock proteins other than PER/TIM. These other proteins could well be CLK and CYC, as loss of these yields morphological defects similar to those seen with reduced NUP153 and mutant RAN [41]. Thus, we believe that only IMPα1 is specific for the nuclear entry of PER/TIM, and other components we tested (nup153 and Ran) have a more general role in clock cells.

We show here that the TIM^PL^ mutant and the TIM^TA^ mutant are defective in their interaction with IMPα1, which most likely accounts for the cytoplasmic localization of these proteins. Given our data showing that the interaction between TIM and IMPα1 is dependent on the TIM NLS, these mutations probably affect the conformation of TIM and block access to the NLS. We hypothesize that phosphorylation at threonine 113, which is also affected by the PL mutation, is required to expose the NLS and allow TIM binding to IMP α1. This suggests that phosphorylation is important for nuclear entry by regulating the interaction of TIM with the nuclear import machinery, thus providing a specific new function for phosphorylation in circadian clock function.

Our cell culture experiments showed that IMPα1 had no effect on PER localization (PER + IMPα1) but dramatically increased nuclear expression of PER/TIM when TIM was added (PER + TIM + IMPα1), supporting the idea that TIM is required for PER nuclear entry. Also, IMPα1 increased nuclear expression of TIM in the absence of PER (TIM + IMPα1), although more nuclear localization of TIM was detected when PER was co-expressed. These results are consistent with the idea that (1) TIM is needed for the nuclear translocation of PER [22,25] and (2) PER retains TIM in the nucleus [12]. Once PER is transported, TIM may not immediately be retained in the nucleus, which would explain the slightly earlier nuclear expression of PER [24]. The requirement of TIM for the nuclear entry of PER is a surprising new twist in the classical nuclear import pathway- the cargo molecule (PER) needs an adapter molecule (TIM) to bind to the classic adapter molecule (IMPα1). We suspect that this unusual modification of the classic import pathway has to do with the need to tightly regulate the nuclear translocation of PER in order to generate a clock.

As mentioned earlier, TIM shuttles in and out of the nucleus, regardless of whether PER is present [12]. While the export is leptomycin-dependent, and therefore likely mediated by exportins, the nuclear import is probably dependent on the pathway we report here. The mutant TIM^PL^ protein is cytoplasmic even in the presence of leptomycin [25], suggesting that lack of an interaction with IMPα1 prevents its shuttling. As noted above, TIM is only retained in the nucleus when PER is nuclear. So is the timing of nuclear expression of TIM-PER, in the middle of the night, controlled only by PER? We do not believe this is the case based upon the phenotype of a new *dbt* mutation (*dbt* ^EY02910^) we recently characterized [42]. In this mutant, PER can be detected in the nucleus at all times, and yet TIM still shows temporal nuclear expression in the presence of light:dark cycles [42]. We suggest that normally TIM, and perhaps PER, is modified in the middle of the night, and subsequently PER is recruited into the TIM-IMPα1 complex and PER and TIM are retained in the nucleus. Prior to this, PER is likely anchored in the cytoplasm by DBT, and even when it is nuclear (in the *dbt* mutant) it cannot retain TIM. The modifications probably include phosphorylation, given the data implicating specific kinases [8,30,43,44] and phosphatases [45] in the timing of nuclear entry. In the absence of DBT, PER may be recruited into the TIM-IMP complex at all times, so it is constantly nuclear, while TIM continues to shuttle until a specific time. Interestingly, PER is even nuclear in the tim^01^; *dbt* ^EY02910^ double mutant flies at ZT21 [42]. Thus, in the absence of DBT and TIM, PER may be transported into the nucleus by non-regulated nuclear import pathways.

At this point, we do not know why TIM is retained in the nucleus given that PER appears to be the major component required for negative feedback [3]. Nevertheless, TIM must have an important function in the nucleus as its nuclear expression is tightly regulated. Loss of circadian cycling of TIM in IMPα1 mutants suggests that clock-controlled degradation of TIM occurs only in the nucleus (note that light-driven cycling can occur in the nucleus or cytoplasm). As maximal phosphorylation of PER as well as maximal repression by it occur only after TIM is degraded at daybreak, TIM may serve to delay maximal feedback. Such delays are thought to be essential for maintaining a clock per se, and also for the circadian timing of it. Thus, TIM may be required to transport PER to the nucleus and to delay feedback by it. Consistent with this idea, the interaction between PER and CLK, which is thought to be critical for transcriptional repression, is TIM-dependent [46]. In addition, the functions of TIM in PER stability [10,47] and in entrainment to light are well-known [48,49]. Together these and other findings are providing a mechanistic, step-by-step account of how a clock might be generated.

## Materials and Methods

### Fly strains and behavioral assays

RNAi stocks were obtained from VDRC and Bloomington stock centers. Df(3L)α1S1, *importin α1* null mutant, flies were kindly provided by Dr. R. Fleming [28]. RanDN flies were kindly provided by Dr. W. Odenwald [50]. The UAS-*importin α1*-HA construct was generated by inserting the coding region of *importin α1* tagged with HA epitope into the pUAST-attB vector. Transgenic fly lines carrying this construct were generated by the site-specific PhiC31 Integration System (Rainbow Transgenics) using the attP on the 2^nd^ chromosome [51]. These transgenic flies were outcrossed 5–6 times into an isogenic w^1118^ (iso^31^) strains. For the behavioral assay, male flies were entrained to 12 h light/dark cycles at 25°C for a least 3 days. Locomotor activity rhythms were measured for 7 to 12 days in DD or LD as previously described [52].

### Whole-mount immunohistochemistry

3–5 day old flies were collected at indicated Zeitgeber times (ZT) on the 4th day of LD entrainment or on the indicated day of LD after drug (RU486 or EtOH) treatment. Fly heads of each genotype were dissected open, and brains were immediately fixed with 4% paraformaldehyde (in 1× PBS) for 15–20 minutes, followed by dissection in 1× PBST (0.3% Triton X-100 in PBS) at room temperature. After a 30min-wash with 1x PBST at room temperature, brains were blocked with 5% normal donkey serum (NDS) for 20 minutes, and then incubated overnight at 4°C with primary antibody: rat anti-PER (UPR34, 1:1000), anti-TIM (UPR42, 1:500), and mouse anti-PDF (C7, Developmental Studies Hybridoma Bank, 1:500). After a 30-min wash in 1× PBST at room temperature, brains were incubated with secondary antibodies (Jackson ImmunoResearch Laboratories, 1:500) for two hours in NDS at room temperature, followed by an extensive 30min-wash. Samples were imaged using a Leica TCS SP5 confocal microscope. Eight to ten brains were examined for each time point.

### Quantitative real-time PCR

Fly heads were collected on dry ice at indicated time points in LD. Total RNA was isolated using the Trizol isolation system (Life Technologies). After DNase treatment, cDNAs were synthesized by using a high-capacity cDNA Reverse Transcription kit (Applied Biosystems). RT-PCR was performed on an ABI prism 7100 using a SYBR Green kit (Applied Biosystems). The sequences of primers used for real-time PCR are as follows: *per* (fwd: 5'- CCAGATTCCCGAACGTCCGT-3'; rev: 5'- GCAGGAGTGGTGACCGAGTG-3'), *tim* (fwd: 5'-CCAATGGACAAAAAGGAGCTTAGA-3'; rev: 5'-GTAACCCTTG AGGAGGAAATCCAC-3'), *importin α1* (fwd: 5'-CCAATGATAAAATCCAGGCT GTAA-3'; rev: 5'-GGCTAATGCAGGT CAAAGCGTTGT-3'), importin α2 (fwd: 5'-GGCACAGATCAACAGACTGACG-3'; rev: 5'- TGCTTCTGGTTACCTGCTGT GA-3'), importin α3 (fwd: 5'- ACCTTGATCAAGGAGGGCG TCATT-3'; rev: 5'- TTCCTCAATGCAGTTGGCCACCGC-3'), importin α4 (fwd: 5'- CAAAATTC GAGCCGACGCCGCAGA-3'; rev: 5'- AATATACCCTTTTCGCAGACTTCA-3'), and *actin* (fwd: 5'-GCGCGGTTACTCTTTCACCA-3'; rev: 5'-ATGTCACG GACGATTTCACG-3').

### Western blot analysis

At the indicated time points in LD or DD, eight to ten fly heads were collected on dry ice and homogenized in EB1 lysis buffer (1X CompleteEDTA-free Protease Inhibitor (Roche), 20mM HEPES pH 7.5, 100mM KCl, 5% glycerol, 2.5mM EDTA, 5mM DTT, 0.1% Triton X-100, 25mM NaF, 0.5mM PMSF) [53]. After 30-minute incubation on ice, fly head extracts were spun down, boiled and resolved on 4–12% Tris-Glycine gels (Novex; Life Technologies). Gels were transferred to nitrocellulose membrane and probed with the following antibodies: guinea pig anti-PER (PA1141, 1:1000), rat anti-TIM (UPR42, 1:1000), and mouse anti-Hsp70 (sigma, 1:5000). Western blot assays were repeated two or three times with similar results.

### S2 cell transfection and scoring of subcellular localization

The PCR-amplified-coding region of *importin α1*-VSV was cloned into a pIZ/V5-His vector (Invitrogen). pCaspeR-hs-*per*-CFP and pCaspeR-hs-*tim*-YFP were used as previously described [9], and also with advice of Taichi Hara (former Sehgal lab member). S2 cells were cultured in a standard Schneider medium and transfected with these constructs as indicated using an Effectene kit (Qiagen) according to manufacturer's protocol. For RNA interference of *importin α1* in S2 cells, the first 400 bp and last 400 bp of a full-length *Drosophila importin α1* cDNA was PCR amplified using standard PCR techniques and primers encoding a T7 transcription promoter sequence in both directions. *In vitro* transcription followed by DNAse I digestion was used to generate double stranded RNA (Ambion), as per manufacturer's protocols. Medium from cells transfected in a 6-well plate was replaced with 1 ml Schneider's medium lacking FBS a day after transfection and 1 ug of each RNA preparation was added to each well. After 1 hour of shaking incubation at room temperature, 1 ml of Schneider's medium with 20% FBS was added to the cells to bring the FBS levels to 10%. After 48 h, cells were treated with heat shock (37°C) for 30 minutes and then fixed 8 h or at indicated times after heat shock induction. S2 cells were scored as previously described [25]. At least 100 cells were scored for each condition in two or three independent blind tests.

### Immunoprecipitation (IP) and GST pull-down assays

For immunoprecipitation assays in S2 cells, cDNAs encoding *per* and *tim* were cloned into the expression vectors pAc-HA and pIZ/V5-His, respectively. NLS mutants for each construct (pAc-*per*_mNLS-HA and pIZ-*tim*_mNLS-V5) were generated by site-directed mutagenesis with the Quick Change mutagenesis kit (Stratagene). The cDNAs of *importin α1* lacking the IBB domain (*importin α1*∆IBB) and wild-type were amplified and tagged with a VSV epitope using PCR and cloned into a pIZ/V5-His vector. Both pIZ-*tim* ^PL^-V5 and pIZ-*tim* ^TA^-V5 were generated as previously described [25]. S2 cells were transfected with various combinations as indicated. After 60 h, cells were collected and lysed in lysis buffer (1X CompleteEDTA-free Protease Inhibitor (Roche), 50 mM Tris-HCl pH 7.5, 150 mM NaCl, 1 mM EDTA, 0.5% Triton X-100). Extracts were incubated with anti-V5 or anti-VSV antibodies at 4°C for 4 h and followed by 2 h-incubation with Protein G or A Dynabeads (Invitrogen). After washing, bound proteins were eluted and subjected to western blot analysis with the following antibodies: rabbit anti-NUP153 antibody (a gift from Dr. M. Capelson [54], 1:1000), mouse anti-V5 (Invitrogen, 1:1000), anti-HA (Sigma, 1:1000) and anti-VSV (Sigma, 1:1000). To perform the immunoprecipitation from fly head extracts, fly heads were homogenized in EB1 lysis buffer. Extracts were incubated overnight with anti-HA beads (sigma) at 4°C and washed three times with lysis buffer. Proteins were eluted and subjected to western blot analysis. For the GST pull-down assay, the FG repeat of Nup153 or Ran (for GDP-bound) or Ran Q69L (for GTP-bound) was cloned into a pGEX-4T-1 vector (GE Healthcare). These GST-fusion proteins were expressed in E. coli and purified using glutathione-Sepharose 4B beads (GE Healthcare). GST-Ran proteins were loaded with GDP or GTP γS using the Ran activation assay kit from New East Biosciences. GST alone (control) or GST-fusion proteins on beads were incubated with TIM-V5 expressed in S2 cells for 4 h at 4°C. After washing, bound TIM-V5 was detected by using a mouse anti-V5 antibody (Invitrogen).

### TIM homology modeling

Secondary structure prediction programs detected two alpha-helical regions in TIM. These two regions were threaded with different homology modeling programs: PHYRE [55], Robetta [56], ITasser [57], pGenThreader [58], HHPred [59] and Raptor X [60].

## Supporting Information

S1 Fig Knockdown efficiency of the different *importin αs* by RNAi. qPCR analysis of total RNA prepared from fly heads of the indicated genotypes. Levels of each *importin α* were normalized to *actin* mRNA and then to levels of that *importin* in TUG/+; dicer/+ flies. Results from three independent experiments are plotted as mean ± SEM (*p < 0.005, **p < 0.0005, by Student's t-test).
(TIF)

S2 Fig Knockdown of IMPα1, but not other importins, affects the nuclear translocation of PER and TIM in LNvs. Five- to six-day-old files expressing various importin α dsRNAs (genotypes of flies are indicated above the panels) in all clock neurons were entrained to LD for 3 days. Brains were dissected and stained with anti-PDF (purple) and-PER (green) antibodies at ZT1 on the 4^th^ day in LD. Eight to ten brains were examined. Impaired PDF projections of l-LNvs in the medulla are indicated with white arrows.
(TIF)

S3 Fig (Related to Fig. 2C) Expression patterns of PER/TIM in s- and l-LNVs under LD and DD. Wild-type and Df(3L)α1S1 mutant flies were collected every 6 hrs in LD (A, C) and in DD (B, D). Whole-mount brains were stained with anti-PER (green; A, B), anti-TIM (cyan; C, D). PDF signal (purple) was used to mark the s- and l-LNvs.
(TIF)

S4 Fig IMPα1 over-expression does not affect rhythms of *importin α* heterozygotes or the periodicity of *per* mutants. IMPα1 was over-expressed in all clock cells using TUG in *importin α* heterozygotes (A), *per* ^L^ (B), and *per* ^S^ (C) mutant flies. Genotypes are indicated on the top of each panel. Average periods (τ) ± SEM of rhythmic flies are shown at the bottom of each panel. Representative activity records are shown.
(TIF)

S5 Fig (Related to Fig. 3B/C) Quantification of TIM protein oscillations in Df(3L)α1S1 and wild-type flies in LD (A) and DD (B). TIM cycling was blunted in Df(3L)α1S1 flies, compared to wild-type flies, in LD and even more severely disrupted in DD. Protein extracts of fly heads at indicated time points were subjected to western blot analysis using antibodies for TIM and a loading control (Hsp70). Relative TIM levels were normalized to the corresponding loading control bands. The quantification depicts mean ± SD from three independent experiments (A) and two independent experiments (B) (**p < 0.01, by Student's t-test).
(TIF)

S6 Fig (Related to Fig. 4) Downregulation of IMPα1 affects nuclear translocation of PER and TIM in S2 cells. (A) S2 cells were transiently transfected with pCaspeR-*per*-*cfp* and pCaspeR-*tim*-*yfp* with dsRNA of *importin α1*. Cells were monitored over a 9 hours period after heat shock induction and then scored as nuclear (blue) and cytoplasmic (orange). The knockdown efficiency of IMPα1 was confirmed by standard immunoblotting methods. The cell lysates were probed with polyclonal rabbit antibody to Importin α1 (a gift from Dr. Bernard Mechler, Deutsches Krebsforschungszentrum). Western blots were performed as previously described at an antibody dilution of 1:1000 [61]. Antibodies to tubulin were obtained from Sigma and used in a 1:10,000 dilution. (B) PER-CFP, TIM-YFP, and dCLK-YFP were imaged using an inverted Olympus IX70 microscope (60X oil objective, 1.42 N.A.), a CFP/YFP/mCherry filter set and dichroic mirror (Chroma), a CCD camera (Photometrics), and an XYZ piezoelectric stage for locating and revisiting multiple cells. mCherry expression demarcates the nucleus and also indicates the outline of the cell.
(TIF)

S7 Fig (Related to Fig. 5) NUP153 is required for the nuclear translocation of PER and TIM. (A) TIM is expressed at the nuclear rim in l-LNvs of wild-type flies. Brains were dissected and stained with TIM (green) and PDF (purple) antibodies at ZT20 on the 4^th^ day in LD. Nuclear envelope association of TIM is indicated with a white arrow. (B) TIM is expressed at the nuclear rim in S2 cells. S2 cells were transiently transfected with pCaspeR-*tim*-*yfp* and pIZ-*importin α1*-VSV. Cells were fixed 8 hrs after heat shock induction and then stained with anti-Lamin antibody (blue) to mark the nuclear envelope in S2 cells. (C) NUP153 downregulation in all clock neurons during adulthood renders flies arrhythmic and causes lethality. For the behavioral assay of flies with reduced NUP153 only as adults, flies were crossed at 25°C and then reared at 18°C until eclosion. The eclosed flies were entrained to LD cycles at 29°C for 4 days and then assayed for locomotor activity under DD at 29°C.
(TIF)

S8 Fig (Related to Fig. 6) TIM is primary cargo for IMPα1 and serves to co-transport PER (A) Nuclear entry of PER and TIM is affected by deletion of the IPP domain on IMPα1 and also by NLS mutation in TIM. S2 cells were transiently transfected with pIZ-*importin α1*-VSV, pIZ-*importin α1*∆IBB-VSV, and various combinations of wild type or NLS mutants of pCaspeR-*per*-*cfp* and pCaspeR-*tim*-*yfp* as indicated.
Cells were fixed 8 hrs after heat shock induction and then scored as nuclear (blue), cytoplasmic (orange), and uniform (both nuclear and cytoplasmic; gray), respectively. At least 100 cells were counted for each condition in two independent experiments. (B) Interaction between IMPα1 and PER/TIM in flies. HA-tagged IMPα1 was immunoprecipitated from head extracts of TUG driven IMPα1-HA flies (TUG>α1-HA) and control flies (α1-HA/+) at indicated time points. The pellets were assayed for expression of PER and TIM. The levels of PER or TIM that co-immunoprecipitated with IMPα1 (dark green bars) were normalized to background levels in the IP control samples that lacked IMPα1 expression (light green bars). The quantification bars indicate the average ± standard deviation (SD) of two independent experiments. (C) TIM is a karyopherin-like protein. Upper left: Secondary structure prediction programs detect two alpha-helical regions (1–275aa and 570–1148aa) in TIM. Here we show the secondary structure prediction from Psi-PRED [62] with the two regions highlighted in blue and red. The nuclear localization sequence is highlighted in green and it abuts the C-terminal alpha-helical region. T113 and P115, which abrogate IMPα1 binding when mutated, are found in the N-terminal alpha-helical region and are also highlighted in green. Upper right: TIM structural model obtained with RaptorX [60]- the two regions modeled are colored corresponding to the secondary structure presented on the left. Bottom: Since TIM is too large for ab intio modeling approaches we threaded the noted TIM sequences with available homology modeling programs. Threading results are shown in the table and include the template (or templates) selected by the programs, region modeled and sequence identity (if available) as well as score to evaluate the obtained model (see below for explanation). All programs predict an alpha-helical ARM/HEAT repeat like region within the 1–275aa region and most predict these repeats for the 570–1148aa region. Intriguingly, most programs selected karyopherin-like molecules as templates. It has to be pointed out that due to the low sequence homology with the templates the model scores obtained with the different programs are marginal at best and should only be regarded as suggestive. The programs utilized were as follows: PHYRE [55]: a confidence score over 90% indicates true homology; Robetta [56]: a score >0.8 would be considered high; ITasser [57]: TM score >0.5 indicates correct topology and <0.17 random similarity; pGenThreader [58]: threading suggests ARM/HEAT repeats with high confidence; HHPred [59]: E value is the average expected number of non-homologous proteins with a score higher than the one obtained for the database match whereas probability includes the secondary structure score as well; Raptor X [60]: Score is the alignment score falling between 0 and the domain sequence length with 0 indicating the worst. A P value is the likelihood of a predicted model being worse than the best of a set of randomly-generated models for this domain; for mainly alpha proteins P value less than 10^-3 is a good indicator.
(TIF)

S9 Fig (Related to Fig. 6C) The *tim* ^PL^ / *tim* ^TA^ mutation affects the interaction between TIM and IMPα1. S2 cells were transfected with pIZ-*tim*-V5 (wt), pIZ-*tim* ^PL^-V5, or pIZ-*tim* ^TA^-V5 in the presence or absence of pIZ-*imp_α1*∆IBB-VSV as indicated. After 60 hours, cells were subjected to IP using an anti-VSV antibody and detected with an anti-V5 antibody. The interaction between TIM^WT/PL/TA^ and empty vector or IMPα1∆IBB was measured by dividing IP signals by corresponding input signals from three independent experiments.
(TIF)

S1 Table Effects of NUP153 downregulation and RanDN expression on free-running circadian locomotor rhythms. (DOCX)
