# Correction: *Drosophila* TIM Binds Importin α1, and Acts as an Adapter to Transport PER to the Nucleus

**DOI:** 10.1371/journal.pgen.1005205

**Published:** 2015-04-27

**Authors:** The PLOS Genetics Staff

The third author, Lino Saez, should be designated as deceased. The publisher apologizes for this error.
