# Supplementary figures and images for: Drosophila TIM Binds Importin α1, and Acts as an Adapter to Transport PER to the Nucleus
Source: PLoS Genet. 2015 Feb 12;11(2):e1004974. doi: 10.1371/journal.pgen.1004974 (PMC4335507; doi:10.1371/journal.pgen.1004974)

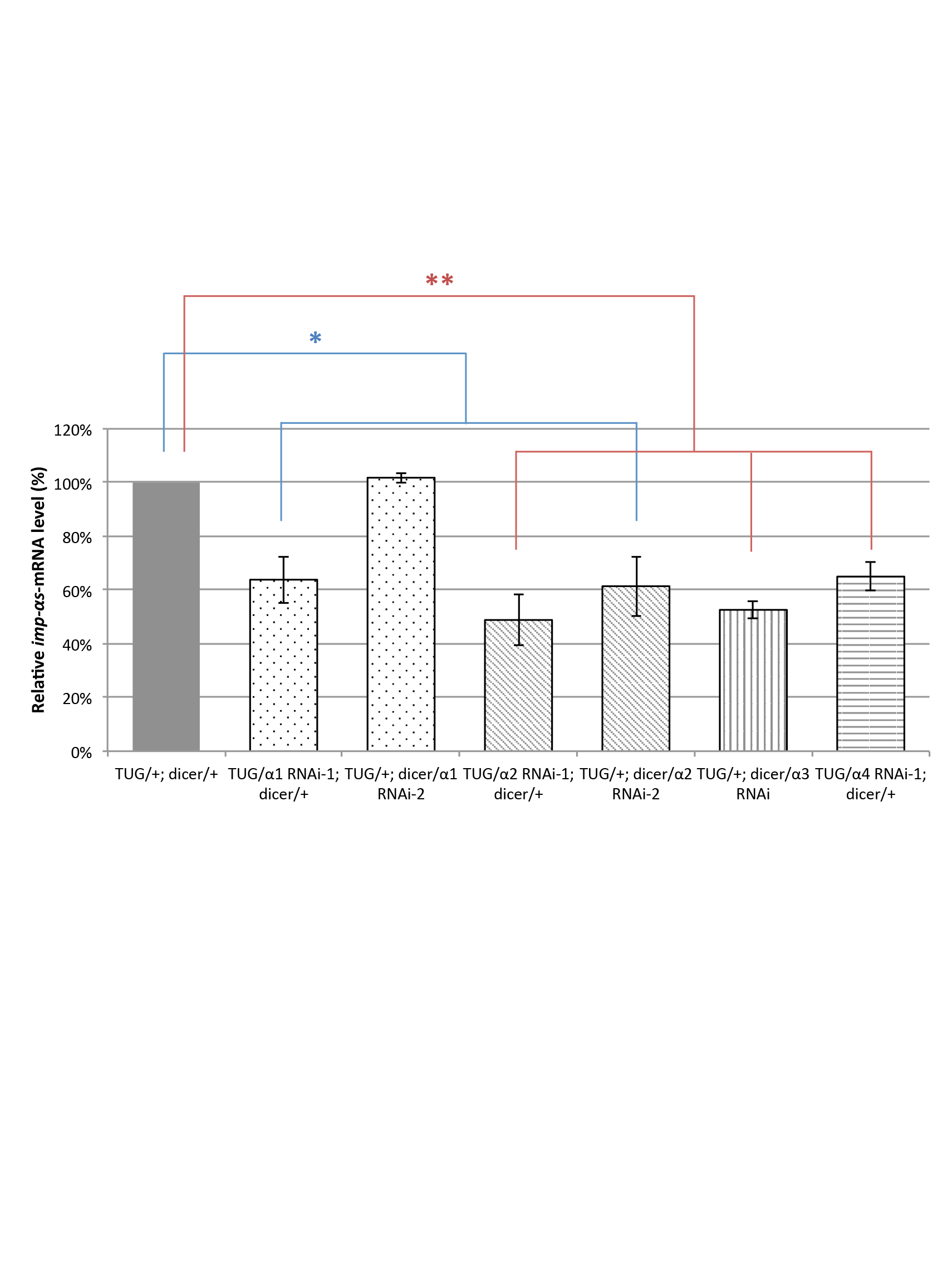

Supplement: S1 Fig — qPCR analysis of total RNA prepared from fly heads of the indicated genotypes. Levels of each importin α were normalized to actin mRNA and then to levels of that importin in TUG/+; dicer/+ flies. Results from three independent experiments are plotted as mean ± SEM (*p < 0.005, **p < 0.0005, by Student's t-test). (TIF) [file pgen.1004974.s001.tif]

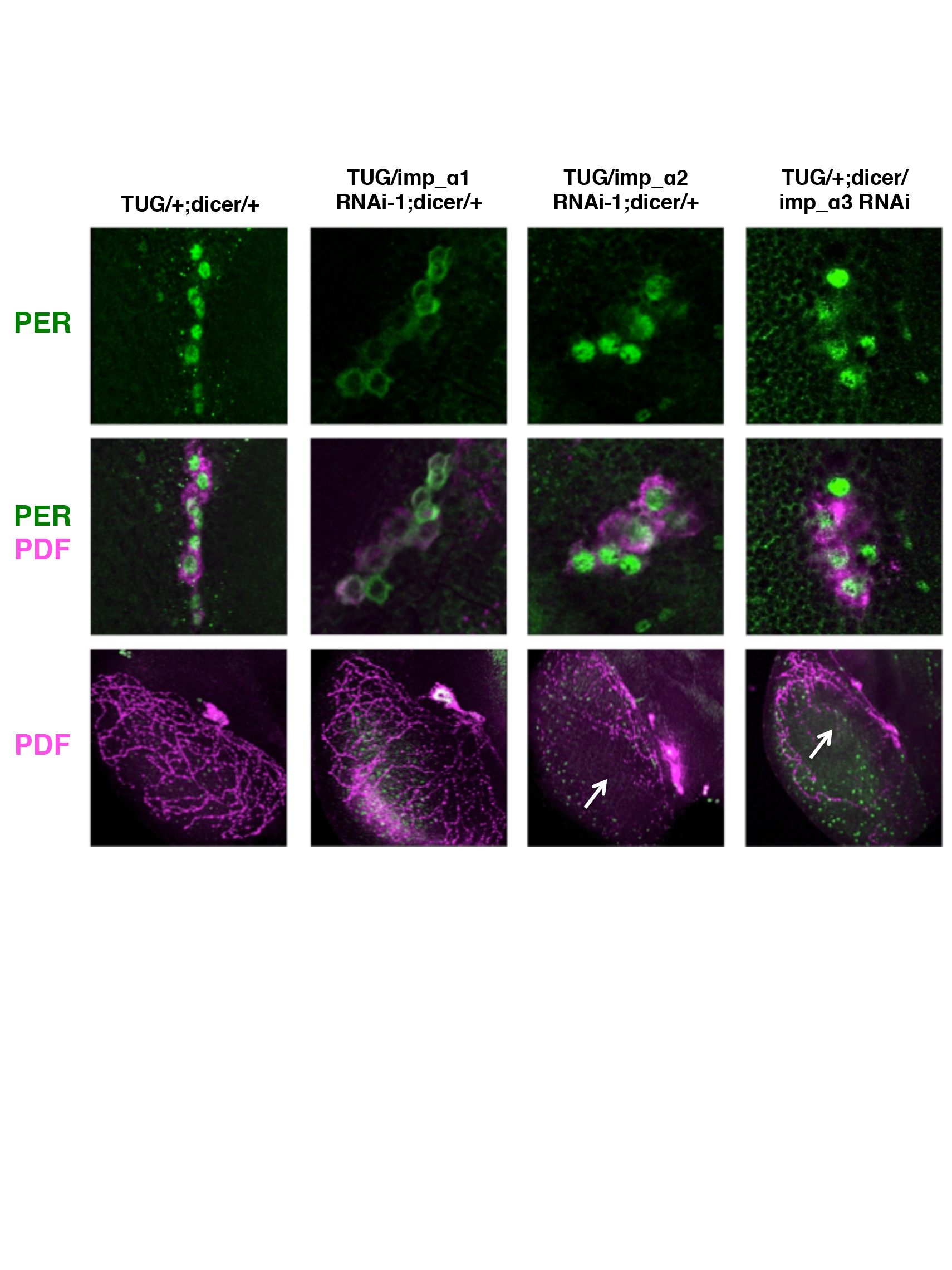

Supplement: S2 Fig — Five- to six-day-old files expressing various importin α dsRNAs (genotypes of flies are indicated above the panels) in all clock neurons were entrained to LD for 3 days. Brains were dissected and stained with anti-PDF (purple) and-PER (green) antibodies at ZT1 on the 4th day in LD. Eight to ten brains were examined. Impaired PDF projections of l-LNvs in the medulla are indicated with white arrows. (TIF) [file pgen.1004974.s002.tif]

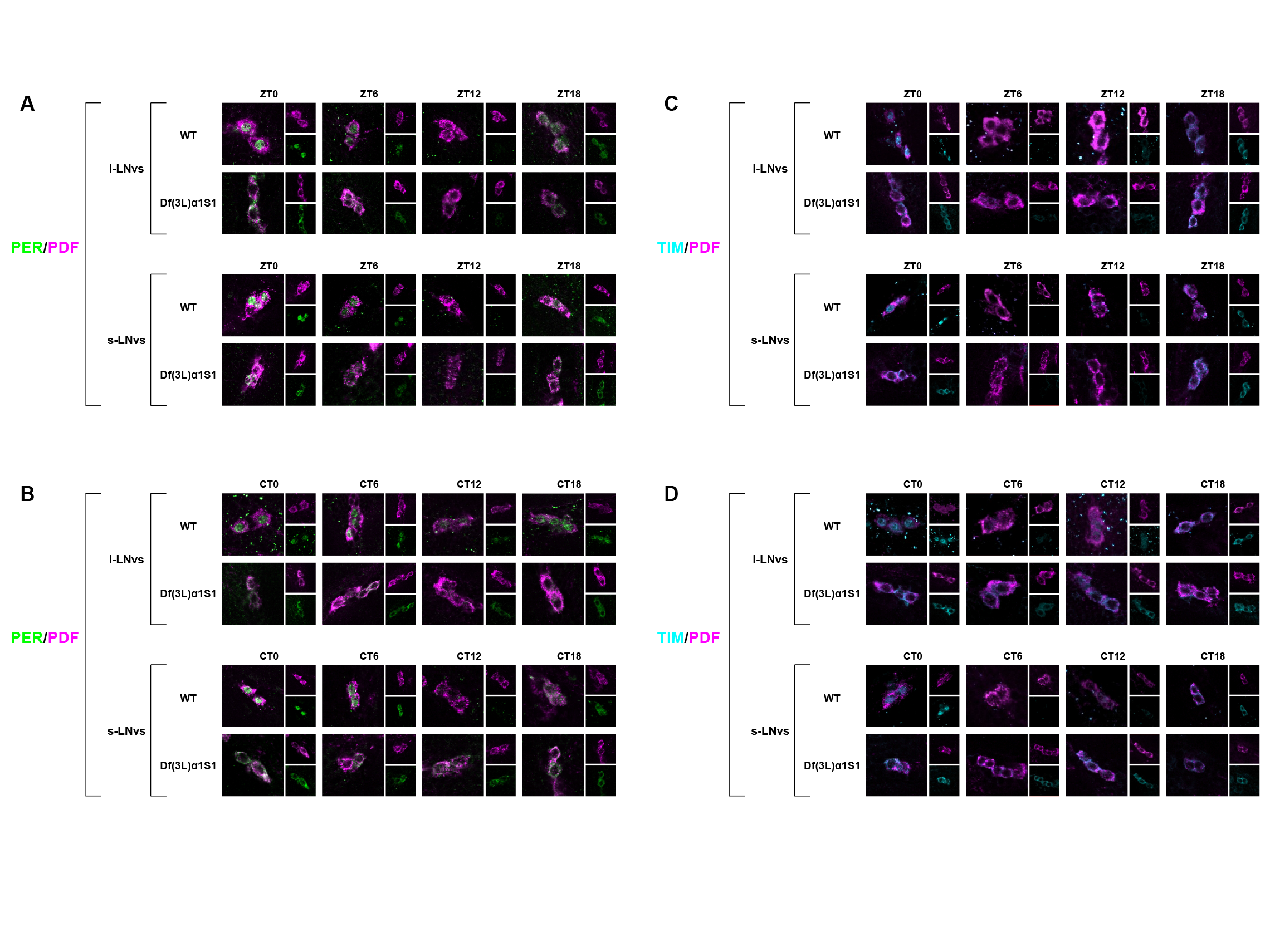

Supplement: S3 Fig — Wild-type and Df(3L)α1S1 mutant flies were collected every 6 hrs in LD (A, C) and in DD (B, D). Whole-mount brains were stained with anti-PER (green; A, B), anti-TIM (cyan; C, D). PDF signal (purple) was used to mark the s- and l-LNvs. (TIF) [file pgen.1004974.s003.tif]

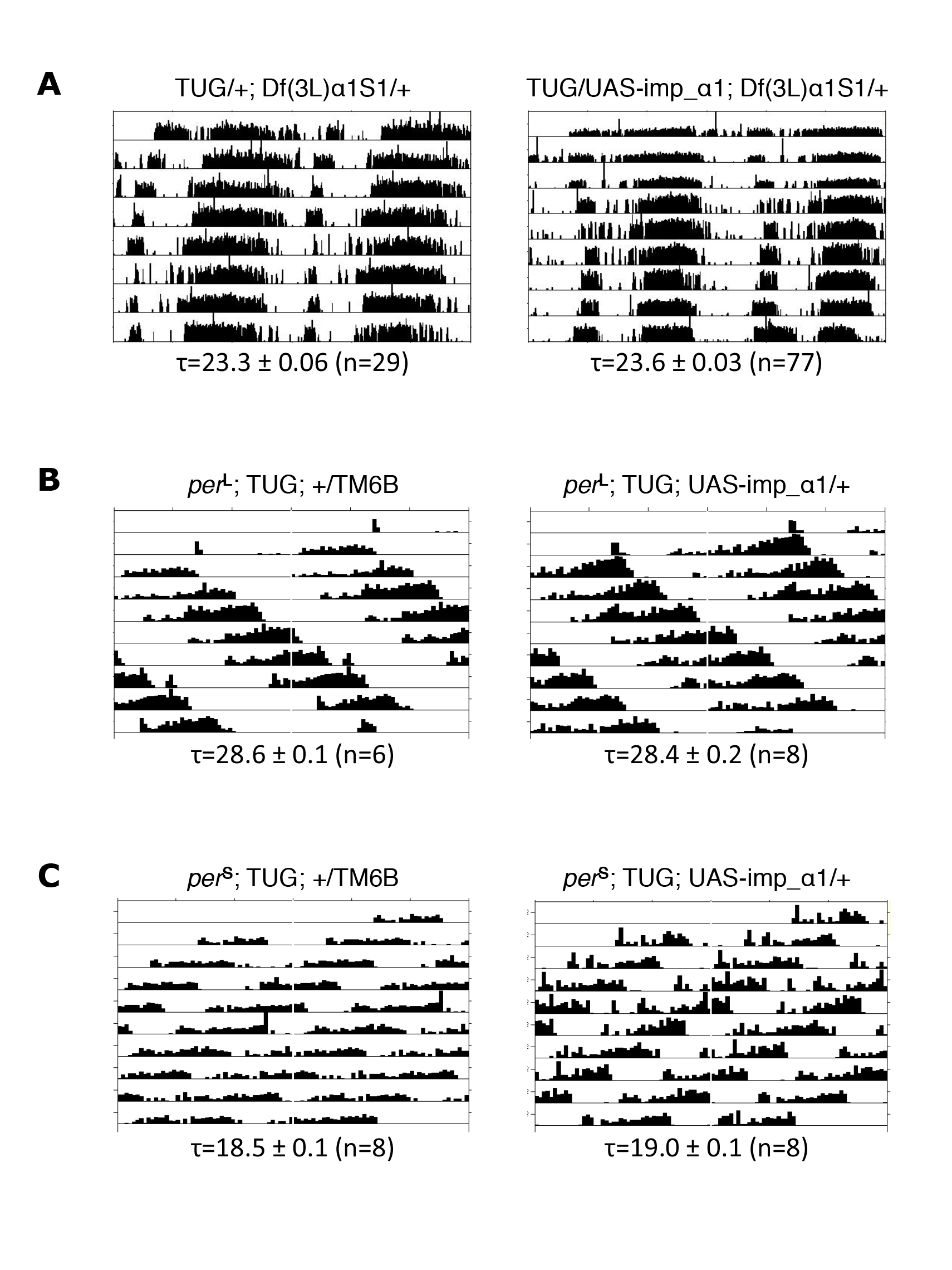

Supplement: S4 Fig — IMPα1 was over-expressed in all clock cells using TUG in importin α heterozygotes (A), per L (B), and per S (C) mutant flies. Genotypes are indicated on the top of each panel. Average periods (τ) ± SEM of rhythmic flies are shown at the bottom of each panel. Representative activity records are shown. (TIF) [file pgen.1004974.s004.tif]

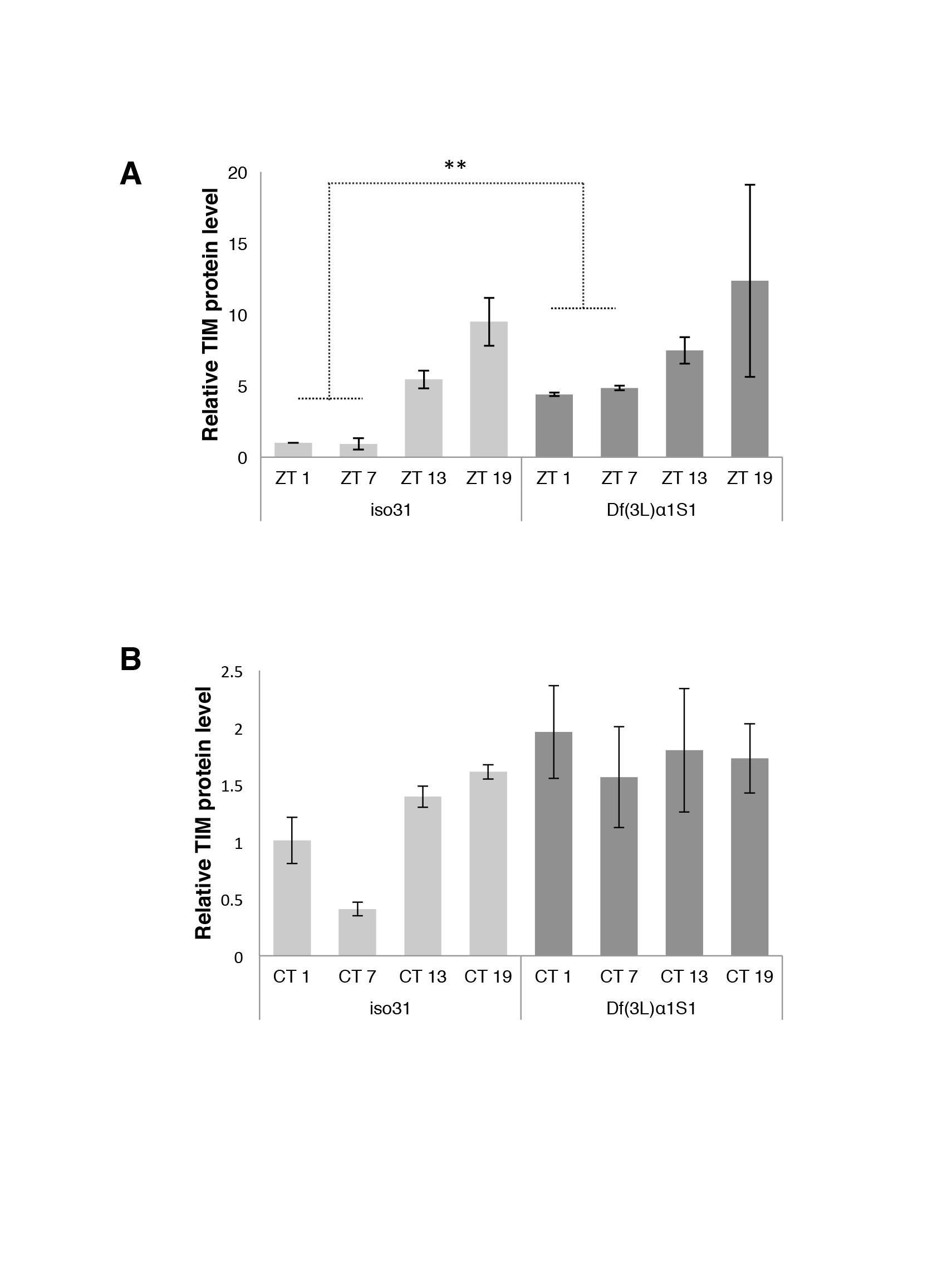

Supplement: S5 Fig — TIM cycling was blunted in Df(3L)α1S1 flies, compared to wild-type flies, in LD and even more severely disrupted in DD. Protein extracts of fly heads at indicated time points were subjected to western blot analysis using antibodies for TIM and a loading control (Hsp70). Relative TIM levels were normalized to the corresponding loading control bands. The quantification depicts mean ± SD from three independent experiments (A) and two independent experiments (B) (**p < 0.01, by Student's t-test). (TIF) [file pgen.1004974.s005.tif]

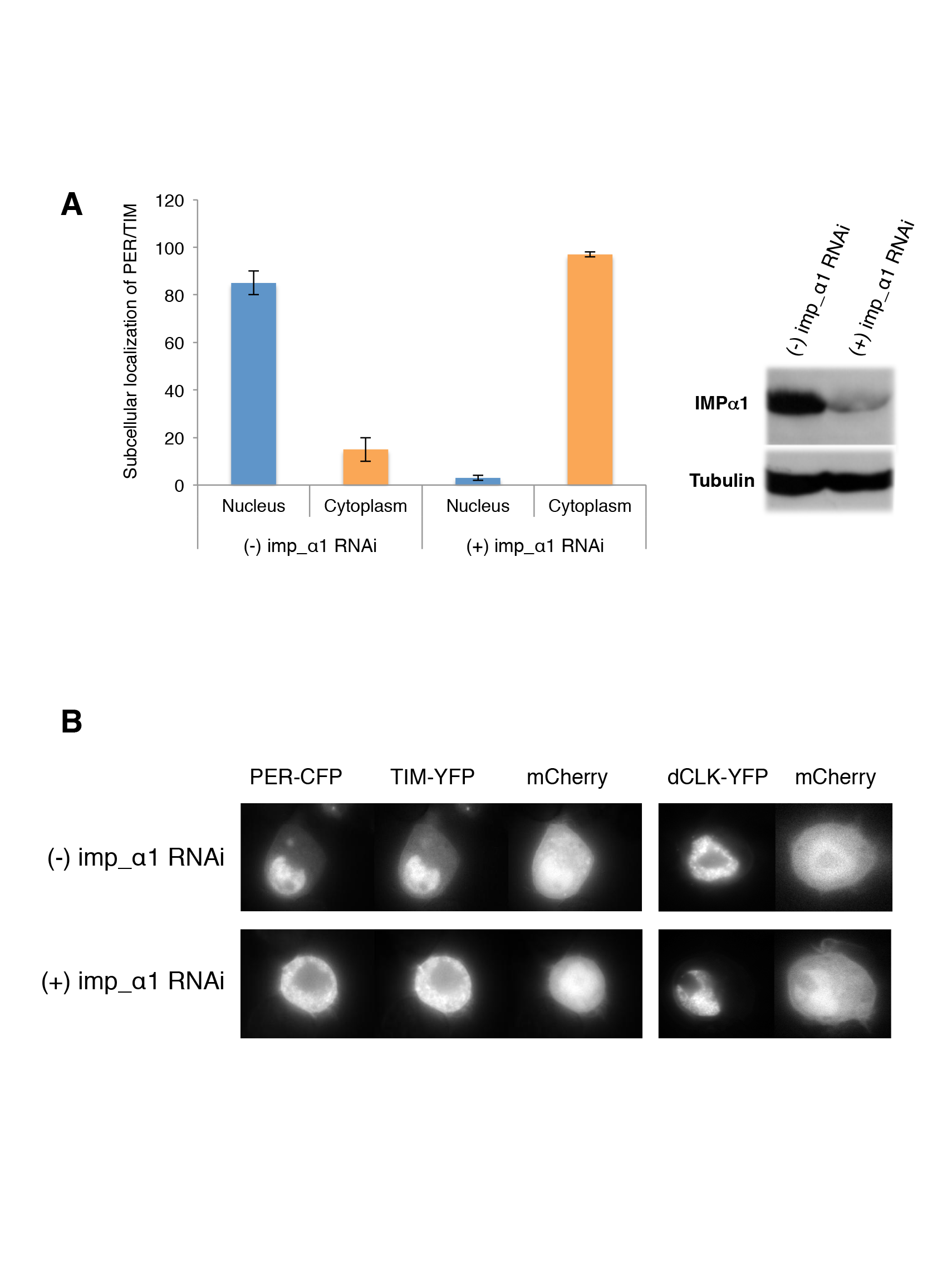

Supplement: S6 Fig — (A) S2 cells were transiently transfected with pCaspeR-per-cfp and pCaspeR-tim-yfp with dsRNA of importin α1. Cells were monitored over a 9 hours period after heat shock induction and then scored as nuclear (blue) and cytoplasmic (orange). The knockdown efficiency of IMPα1 was confirmed by standard immunoblotting methods. The cell lysates were probed with polyclonal rabbit antibody to Importin α1 (a gift from Dr. Bernard Mechler, Deutsches Krebsforschungszentrum). Western blots were performed as previously described at an antibody dilution of 1:1000 [61]. Antibodies to tubulin were obtained from Sigma and used in a 1:10,000 dilution. (B) PER-CFP, TIM-YFP, and dCLK-YFP were imaged using an inverted Olympus IX70 microscope (60X oil objective, 1.42 N.A.), a CFP/YFP/mCherry filter set and dichroic mirror (Chroma), a CCD camera (Photometrics), and an XYZ piezoelectric stage for locating and revisiting multiple cells. mCherry expression demarcates the nucleus and also indicates the outline of the cell. (TIF) [file pgen.1004974.s006.tif]

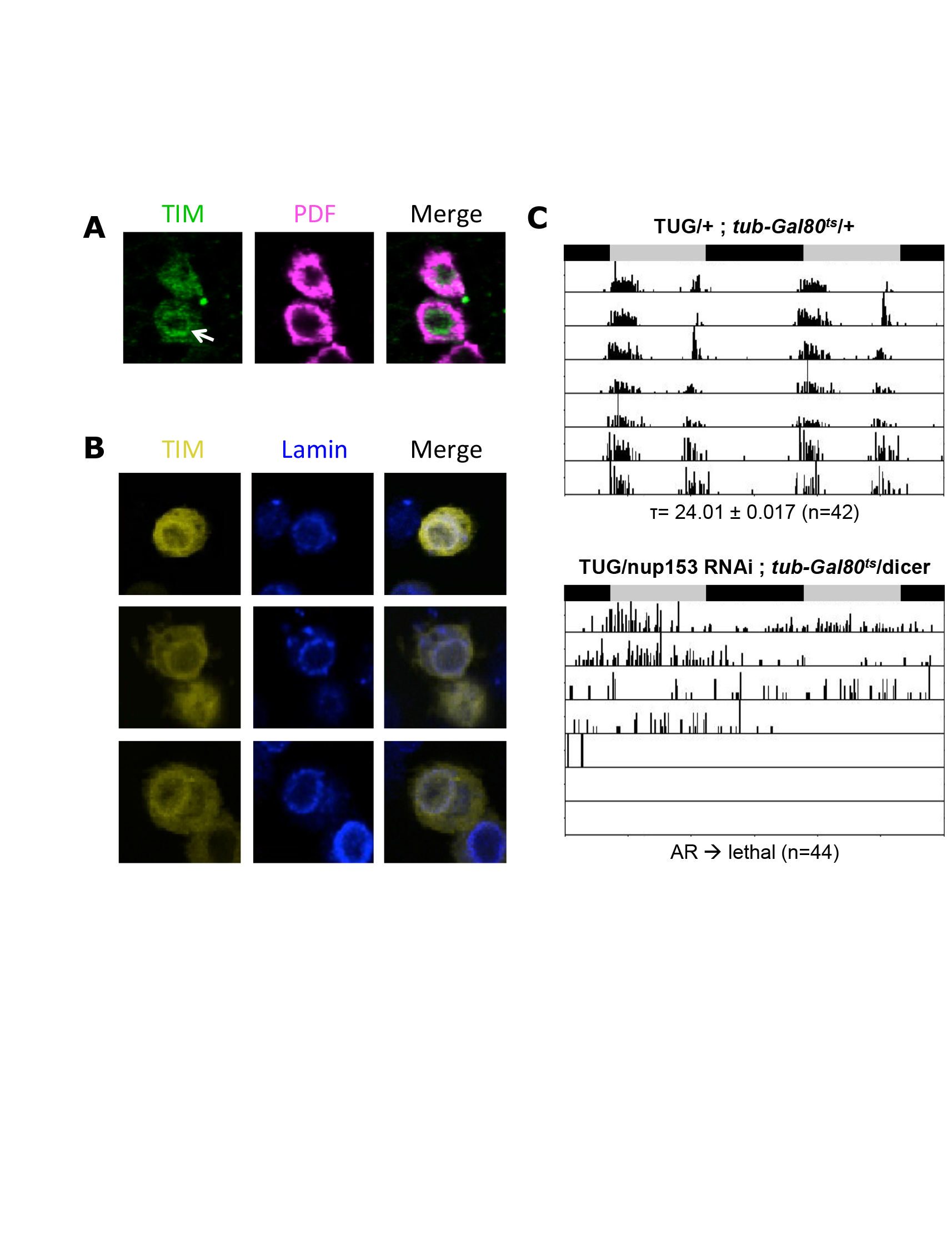

Supplement: S7 Fig — (A) TIM is expressed at the nuclear rim in l-LNvs of wild-type flies. Brains were dissected and stained with TIM (green) and PDF (purple) antibodies at ZT20 on the 4th day in LD. Nuclear envelope association of TIM is indicated with a white arrow. (B) TIM is expressed at the nuclear rim in S2 cells. S2 cells were transiently transfected with pCaspeR-tim-yfp and pIZ-importin α1-VSV. Cells were fixed 8 hrs after heat shock induction and then stained with anti-Lamin antibody (blue) to mark the nuclear envelope in S2 cells. (C) NUP153 downregulation in all clock neurons during adulthood renders flies arrhythmic and causes lethality. For the behavioral assay of flies with reduced NUP153 only as adults, flies were crossed at 25°C and then reared at 18°C until eclosion. The eclosed flies were entrained to LD cycles at 29°C for 4 days and then assayed for locomotor activity under DD at 29°C. (TIF) [file pgen.1004974.s007.tif]

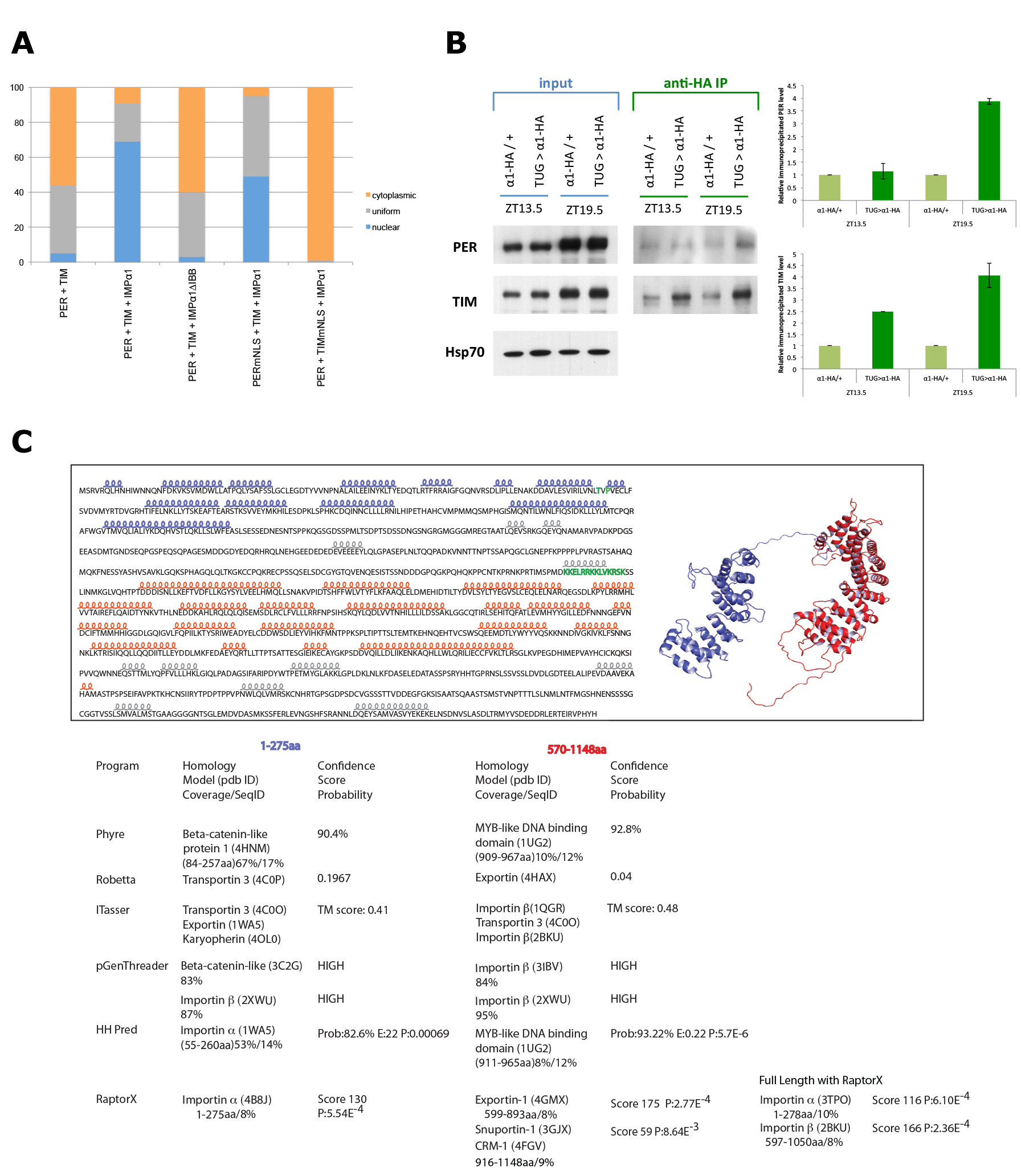

Supplement: S8 Fig — S2 cells were transiently transfected with pIZ-importin α1-VSV, pIZ-importin α1∆IBB-VSV, and various combinations of wild type or NLS mutants of pCaspeR-per-cfp and pCaspeR-tim-yfp as indicated. Cells were fixed 8 hrs after heat shock induction and then scored as nuclear (blue), cytoplasmic (orange), and uniform (both nuclear and cytoplasmic; gray), respectively. At least 100 cells were counted for each condition in two independent experiments. (B) Interaction between IMPα1 and PER/TIM in flies. HA-tagged IMPα1 was immunoprecipitated from head extracts of TUG driven IMPα1-HA flies (TUG>α1-HA) and control flies (α1-HA/+) at indicated time points. The pellets were assayed for expression of PER and TIM. The levels of PER or TIM that co-immunoprecipitated with IMPα1 (dark green bars) were normalized to background levels in the IP control samples that lacked IMPα1 expression (light green bars). The quantification bars indicate the average ± standard deviation (SD) of two independent experiments. (C) TIM is a karyopherin-like protein. Upper left: Secondary structure prediction programs detect two alpha-helical regions (1–275aa and 570–1148aa) in TIM. Here we show the secondary structure prediction from Psi-PRED [62] with the two regions highlighted in blue and red. The nuclear localization sequence is highlighted in green and it abuts the C-terminal alpha-helical region. T113 and P115, which abrogate IMPα1 binding when mutated, are found in the N-terminal alpha-helical region and are also highlighted in green. Upper right: TIM structural model obtained with RaptorX [60]- the two regions modeled are colored corresponding to the secondary structure presented on the left. Bottom: Since TIM is too large for ab intio modeling approaches we threaded the noted TIM sequences with available homology modeling programs. Threading results are shown in the table and include the template (or templates) selected by the programs, region modeled and sequence identity (if available) as wel [file pgen.1004974.s008.tif]

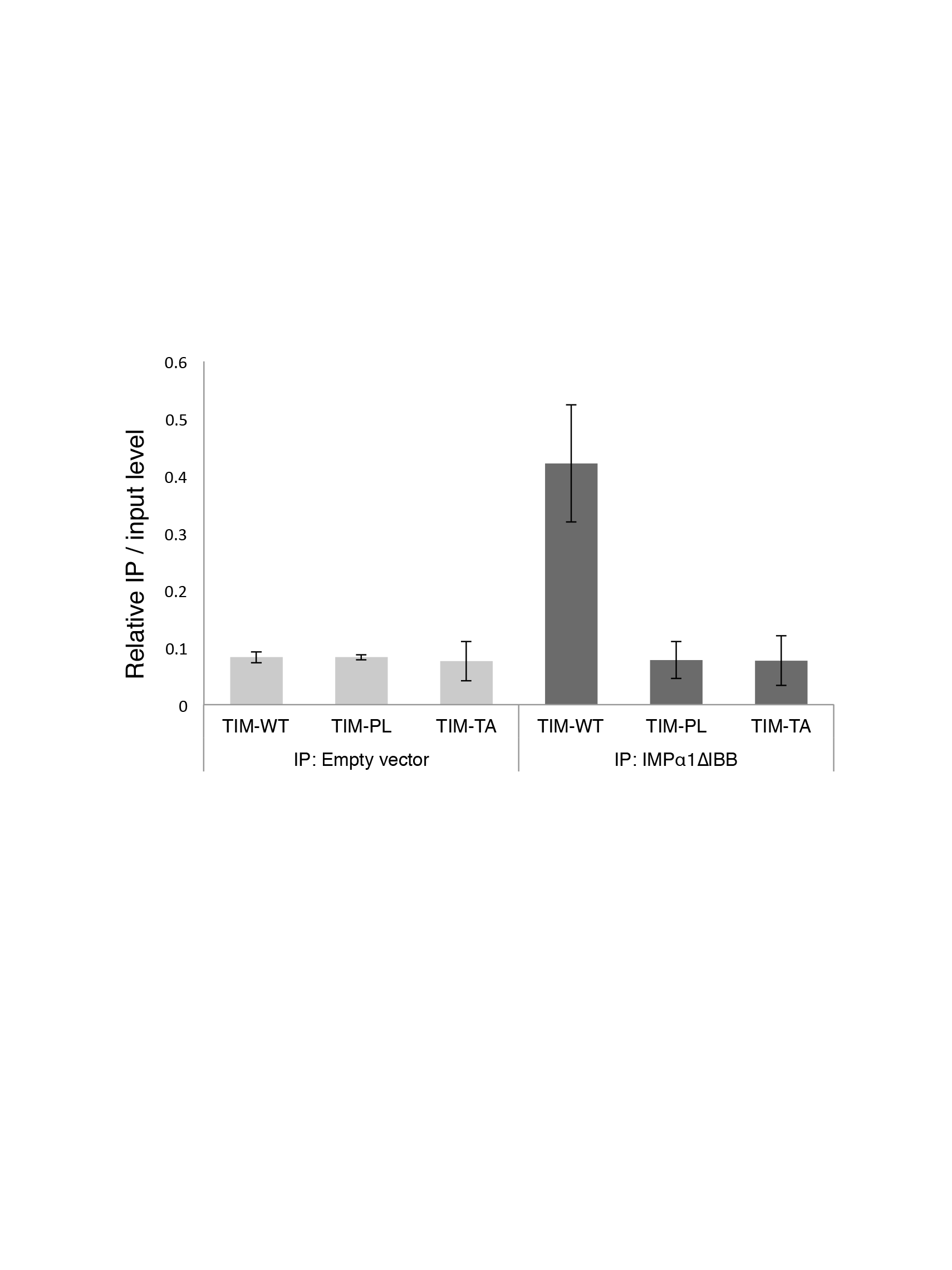

Supplement: S9 Fig — S2 cells were transfected with pIZ-tim-V5 (wt), pIZ-tim PL-V5, or pIZ-tim TA-V5 in the presence or absence of pIZ-imp_α1∆IBB-VSV as indicated. After 60 hours, cells were subjected to IP using an anti-VSV antibody and detected with an anti-V5 antibody. The interaction between TIMWT/PL/TA and empty vector or IMPα1∆IBB was measured by dividing IP signals by corresponding input signals from three independent experiments. (TIF) [file pgen.1004974.s009.tif]
