# Supplementary material for: Drosophila TIM Binds Importin α1, and Acts as an Adapter to Transport PER to the Nucleus
Source: PLoS Genet. 2015 Feb 12;11(2):e1004974. doi: 10.1371/journal.pgen.1004974 (PMC4335507; doi:10.1371/journal.pgen.1004974)
Supplement: S1 Table — (DOCX) [file pgen.1004974.s010.docx]

| **Table S1.**  Effects of NUP153 downregulation and RanDN expression on free-running circadian locomotor rhythms | | | | |
| --- | --- | --- | --- | --- |
|  | | | | |
| **Genotype** | **n** | **Rhythmic**  **Flies (%)** | **Period**  **(hr) ± SEM** | **Power**  **(FFT) ± SEM** |
| *Pdf*-GAL4/+ ; dicer/+ | 12 | 100 | 23.77 ± 0.06 | 0.056 ± 0.010 |
| *Pdf*-GAL4/nup153 RNAi ; dicer/+ | 15 | 33.3 | 23.43 ± 0.04 | 0.019 ± 0.003 |
| *Pdf*-GS > UAS-RanDN (**+ EtOH)** | 36 | 87.5 | 23.81 ± 0.07 | 0.063 ± 0.006 |
| *Pdf*-GS > UAS-RanDN (**+ RU486)** | 41 | 19.5 | 23.57 ± 0.19 | 0.017 ± 0.001 |
